# Supplementary material for: Synthesis, Structural Characterization and Anticancer Activity of New 5-Trifluoromethyl-2-thioxo-thiazolo[4,5-d]pyrimidine Derivatives
Source: Pharmaceuticals (Basel). 2022 Jan 13;15(1):92. doi: 10.3390/ph15010092 (PMC8778818; doi:10.3390/ph15010092)
Supplement: Supplementary file 1 [file pharmaceuticals-15-00092-s001.zip › pharmaceuticals-1515204-supplementary.pdf]

Supporting information

Synthesis, Structural Characterization and Anticancer Activity of New 5-trifluoromethyl-2-thioxo-thiazolo[4,5-*d*]pyrimidine derivatives

Lilianna Becan [1,*], Anna Pyra [2], Nina Rembiałkowska [3] and Iwona Bryndal [1,*]

Table of contents

Figures S1-S15. ^{1}H NMR spectra of compounds **1c**, **1d**, **2a**-**2e**, **3a**-**e**, **4a**-**c**..S2-S9

Figures S16-S28. ^{13}C NMR spectra of compounds **2a**-**2e**, **3a**-**e**, **4a**-**c**..S9-S15

Figures S29-S41. MS spectra of compounds **2a**-**2e**, **3a**-**e**, **4a**-**c**..S16-S20

Figures S42-S54. IR spectra of compounds **2a**-**2e**, **3a**-**e**, **4a**-**c**..S20-S26

Figure S55. The crystal packing of the **2e** viewed along a axis. Disordered toluene molecules are marked with a purple wire/stick model. Dashed lines (in black) indicate intermolecular interactions, with N—H···O in black, C−H···S in orange and C−H···F in light green..S27

Table S1. Selected X-ray single-crystal data and structure refinement details of compounds **2e** and **4b**............S28

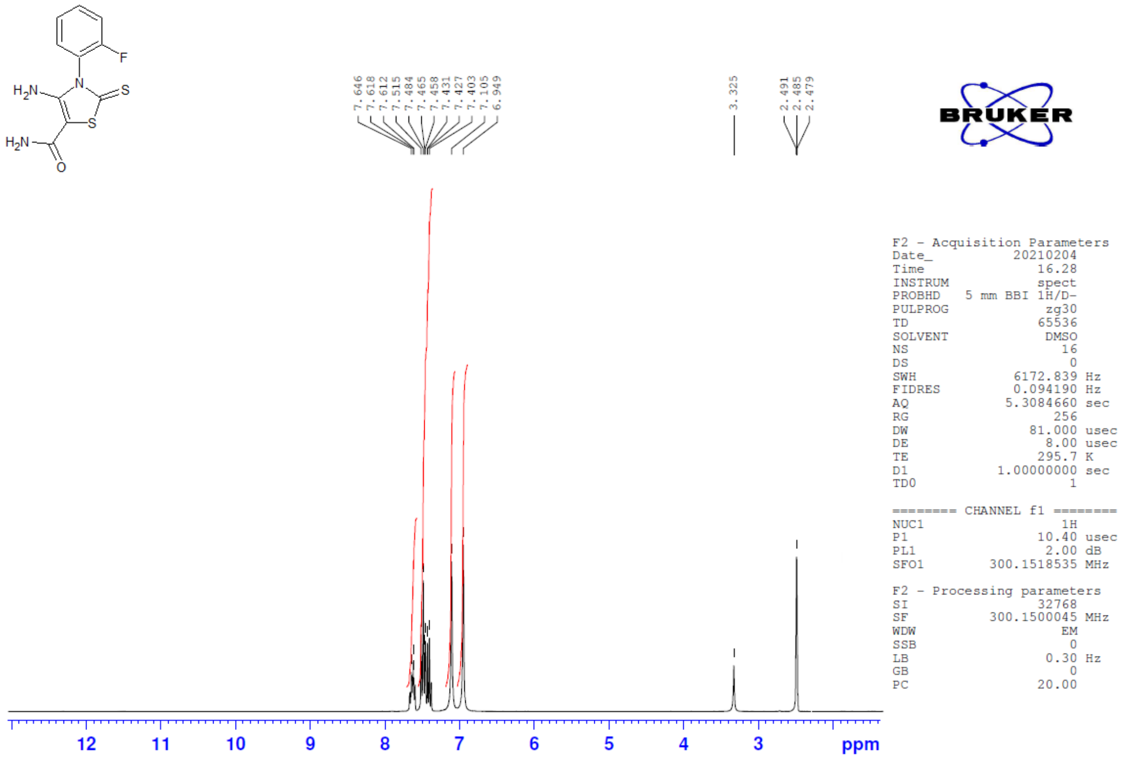

Figure S1. ^{1}HNMR spectrum of compound **1c** (300MHz, DMSO).

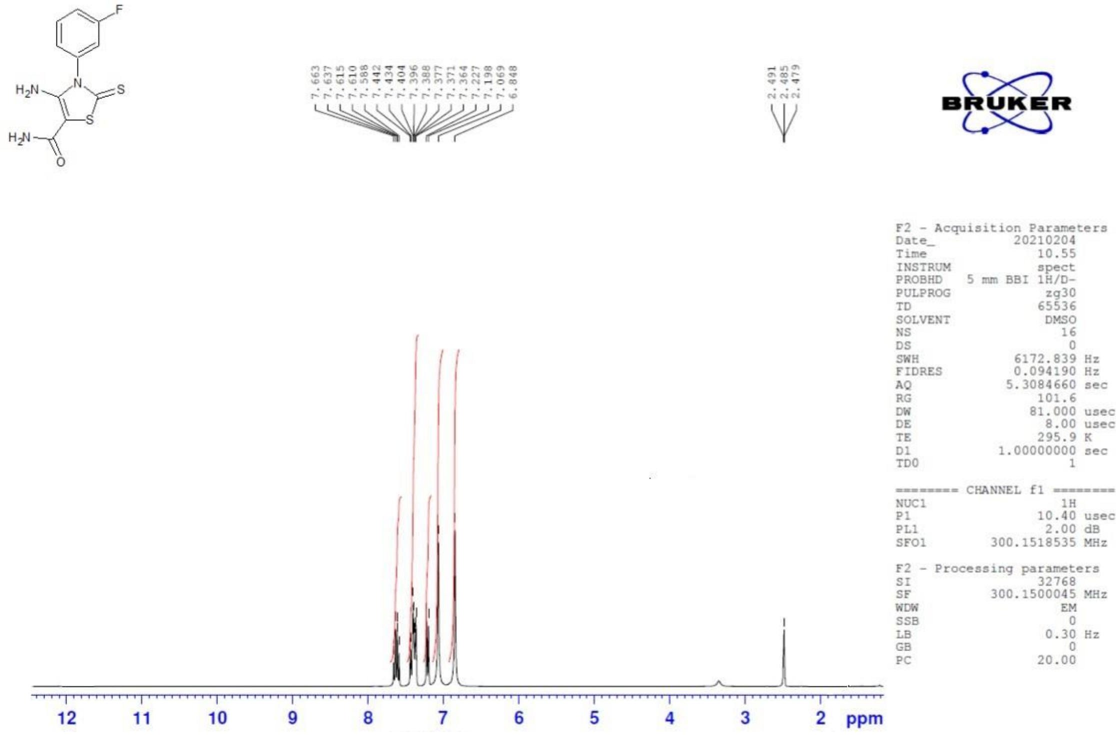

Figure S2. ^{1}HNMR spectrum of compound **1d** (300MHz, DMSO).

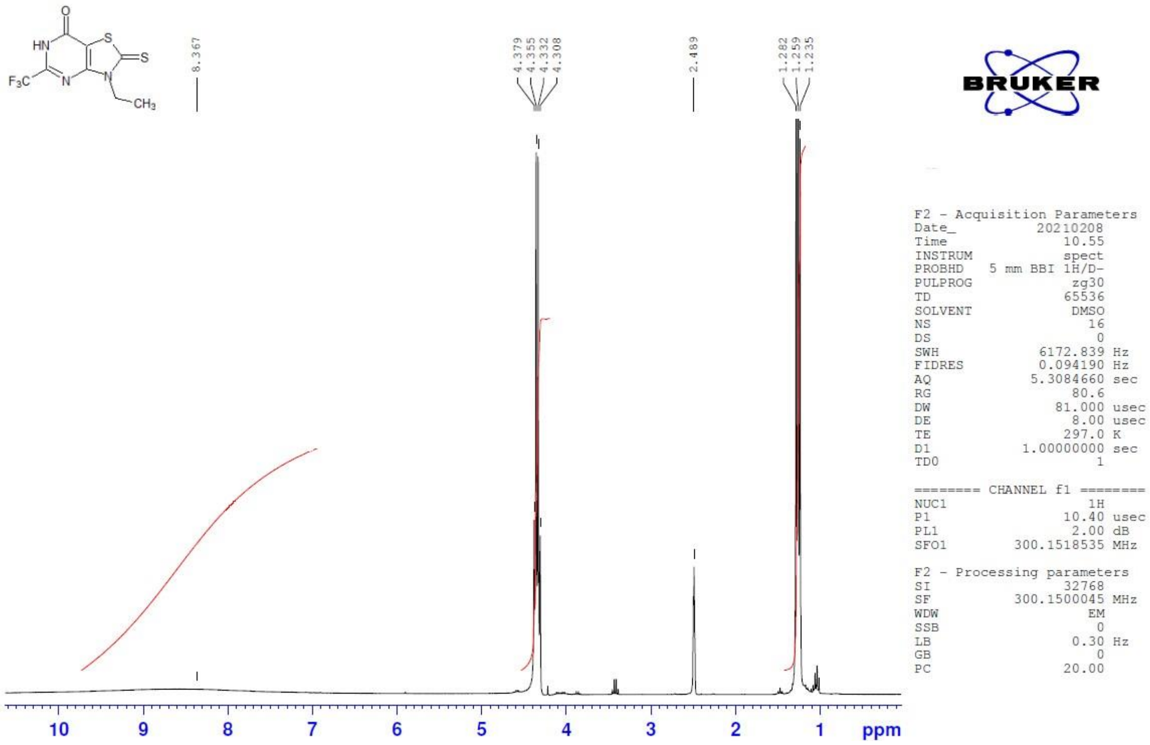

Figure S3. ^{1}HNMR spectrum of compound **2a** (300MHz, DMSO).

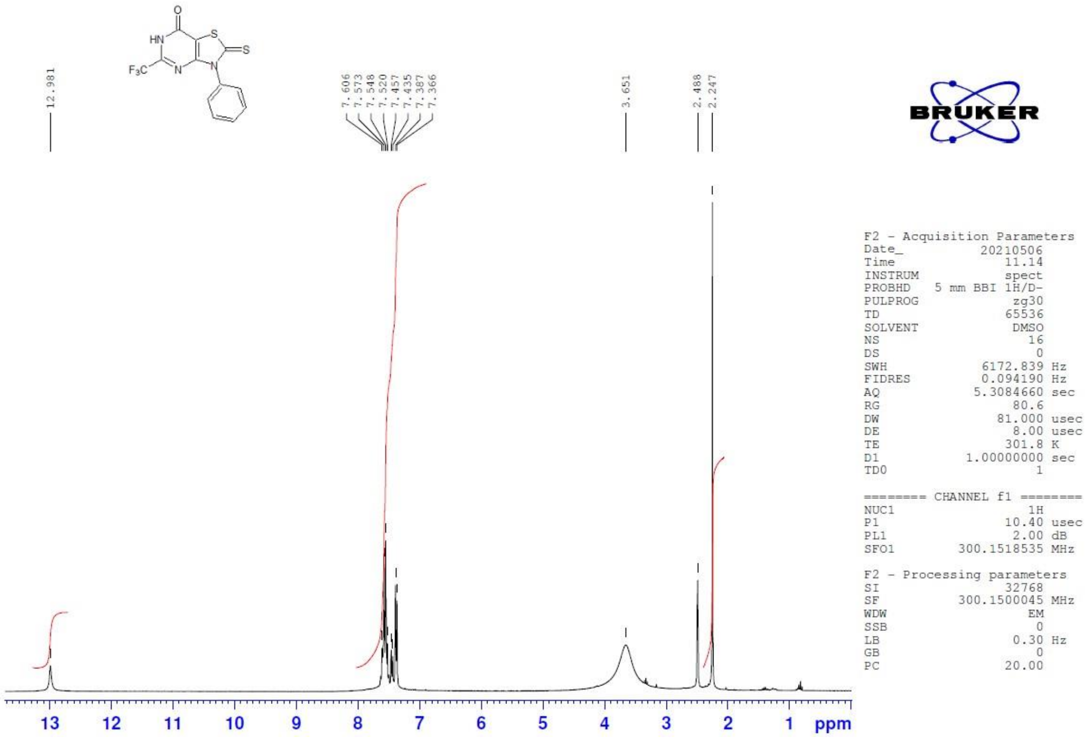

Figure S4. ^{1}HNMR spectrum of compound **2b** (300MHz, DMSO).

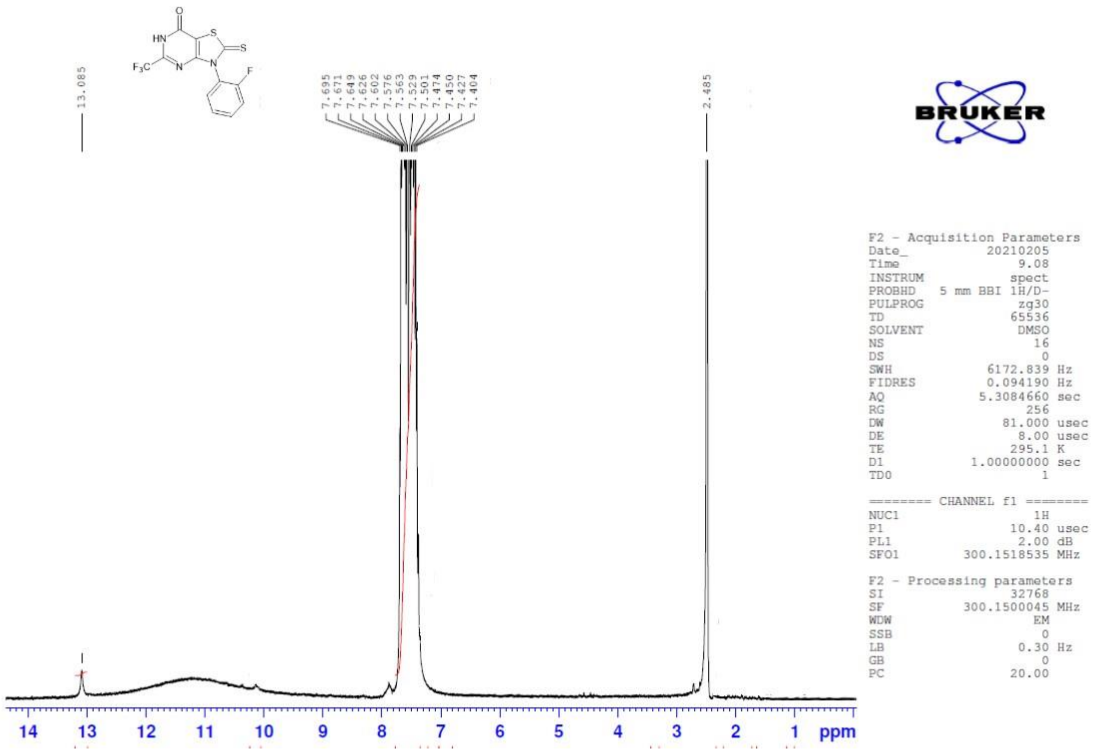

Figure S5. ^{1}HNMR spectrum of compound **2c** (300MHz, DMSO).

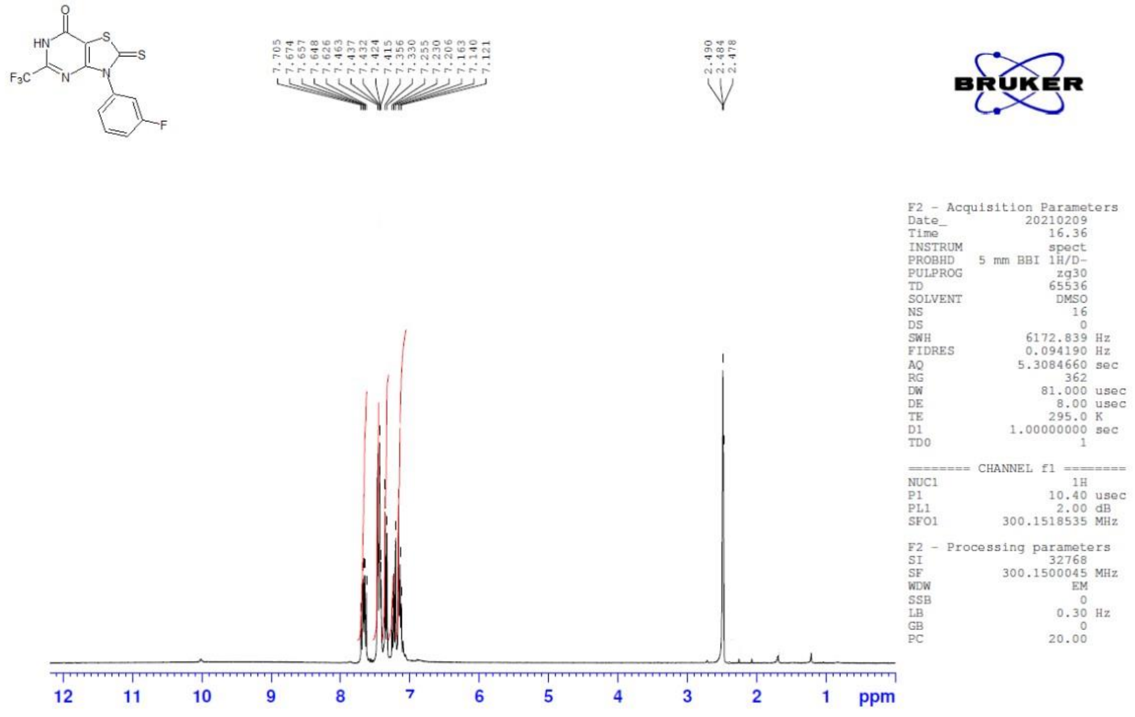

Figure S6. ^{1}HNMR spectrum of compound **2d** (300MHz, DMSO).

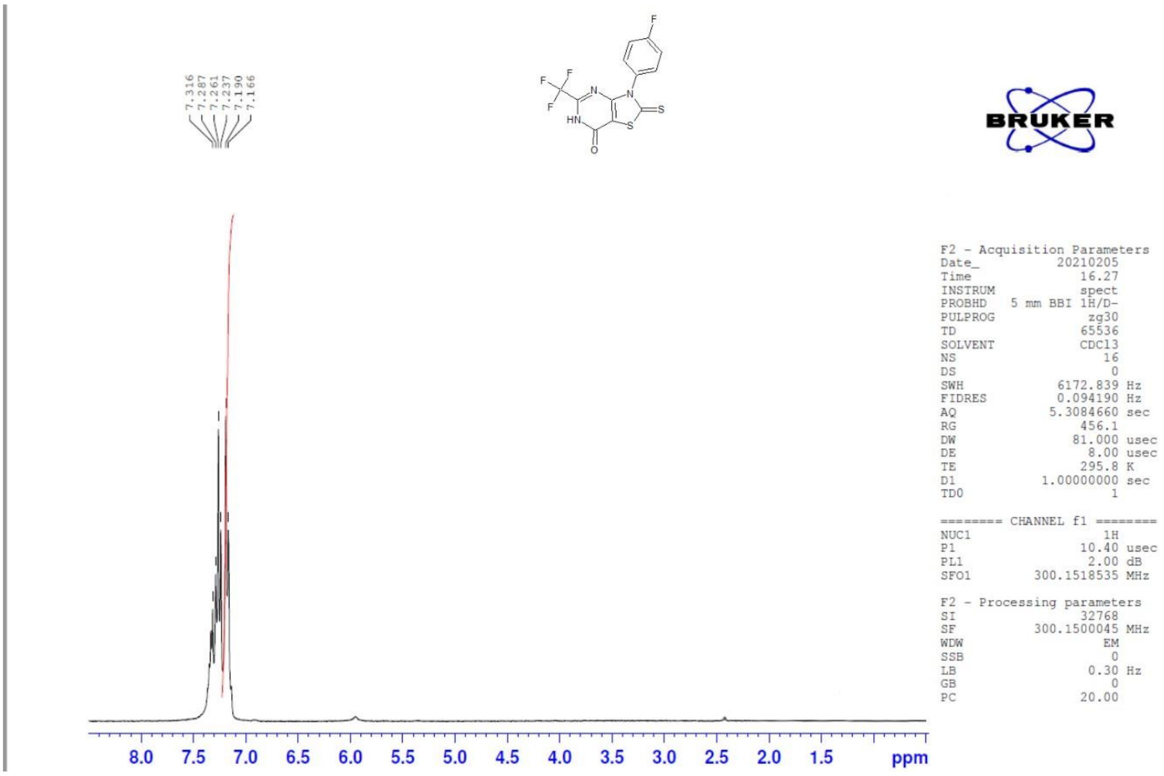

Figure S7. ^{1}HNMR spectrum of compound **2e** (300MHz, CDCl3).

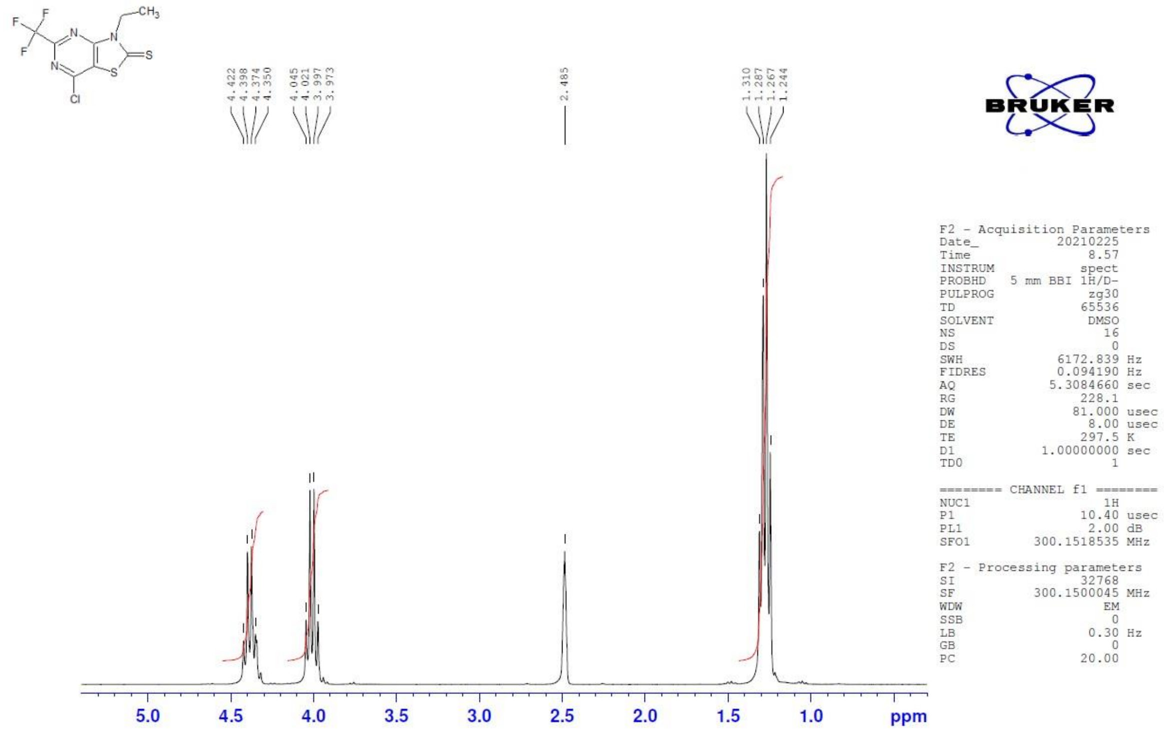

Figure S8. ^{1}HNMR spectrum of compound **3a** (300MHz, DMSO).

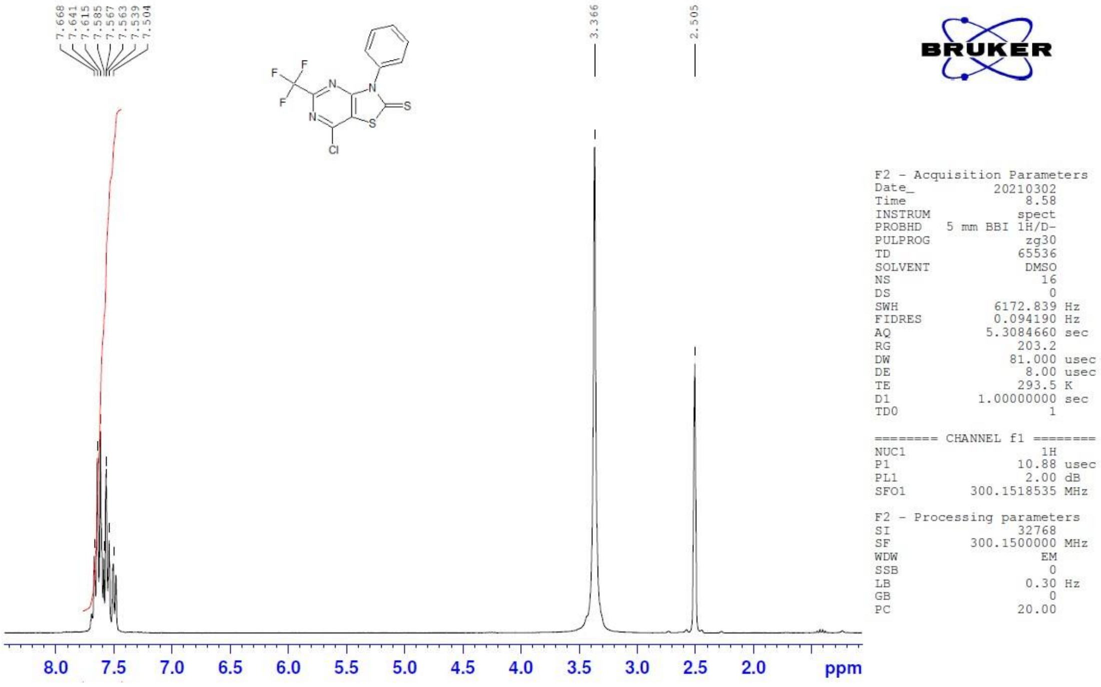

Figure S9. ^{1}HNMR spectrum of compound **3b** (300MHz, DMSO).

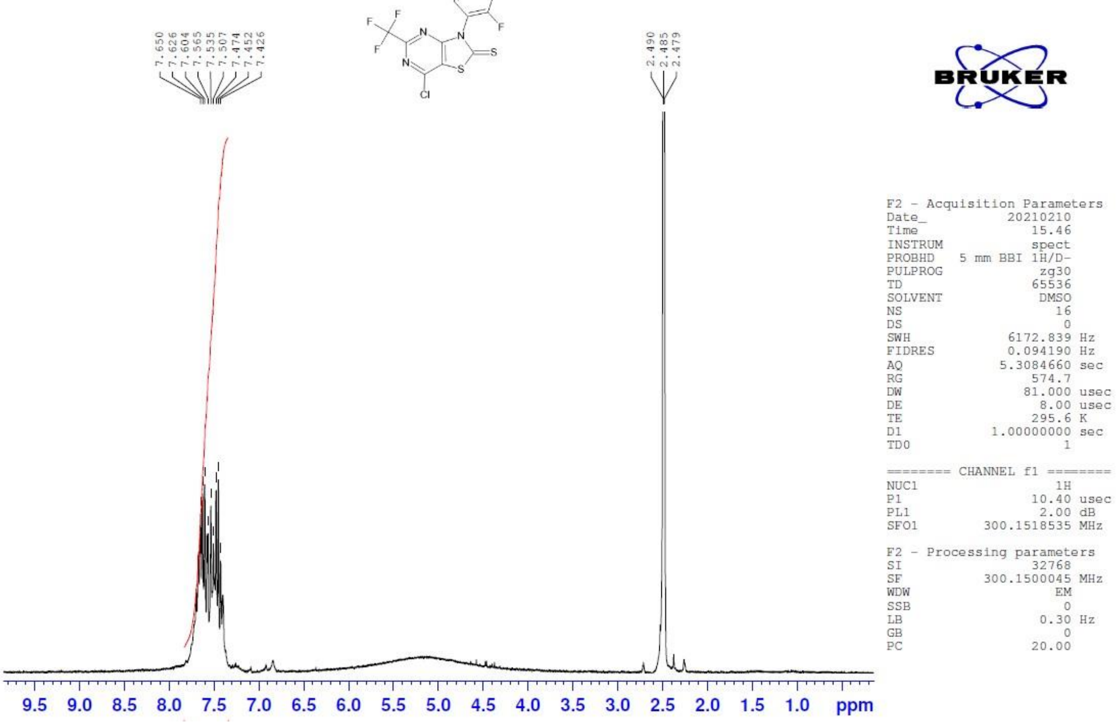

Figure S10. ^{1}HNMR spectrum of compound **3c** (300MHz, DMSO).

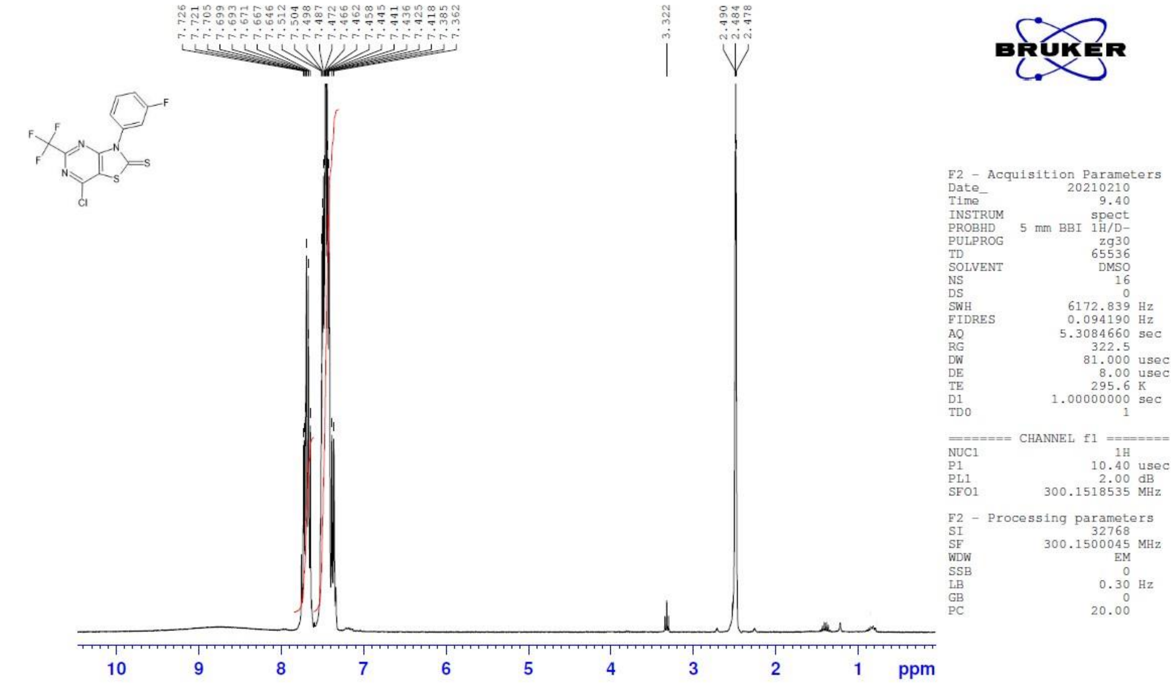

Figure S11. ^{1}HNMR spectrum of compound **3d** (300MHz, DMSO).

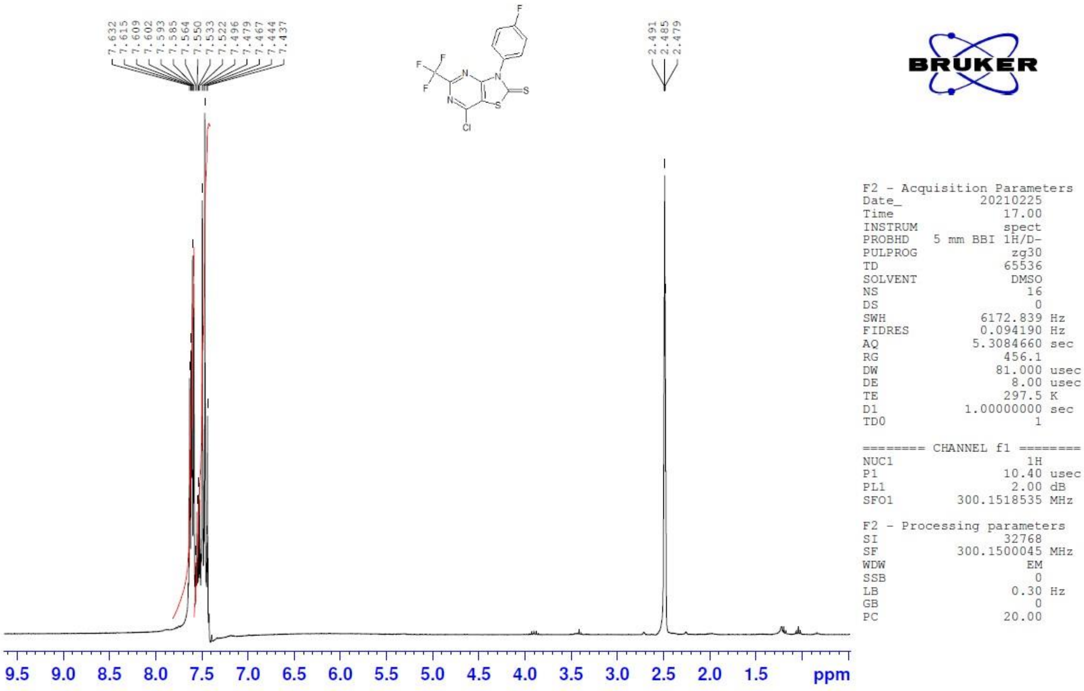

Figure S12. ^{1}HNMR spectrum of compound **3e** (300MHz, DMSO).

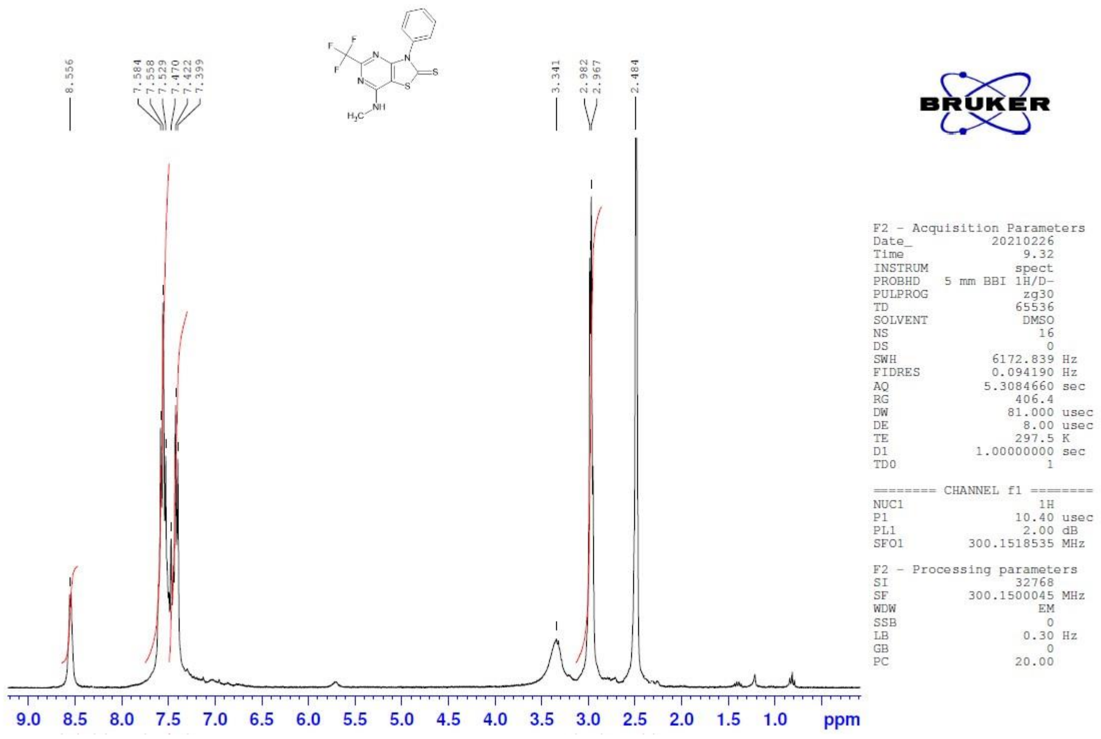

Figure S13. ¹HNMR spectrum of compound **4a** (300MHz, DMSO).

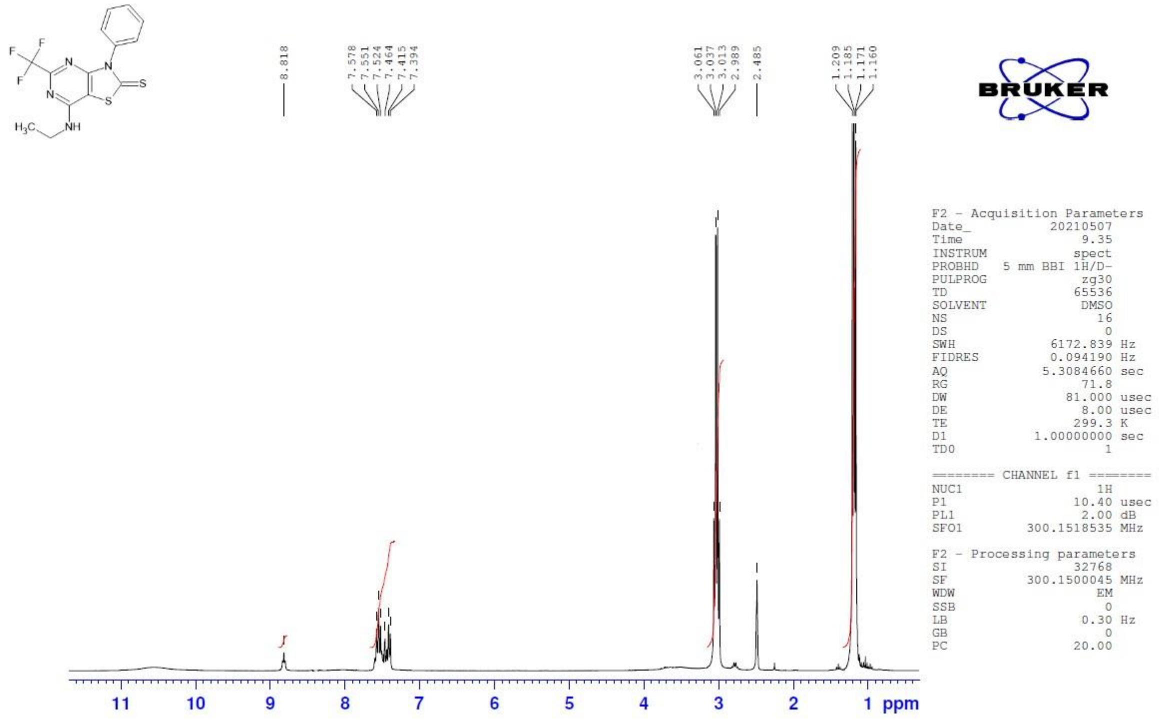

Figure S14. ¹HNMR spectrum of compound **4b** (300MHz, DMSO).

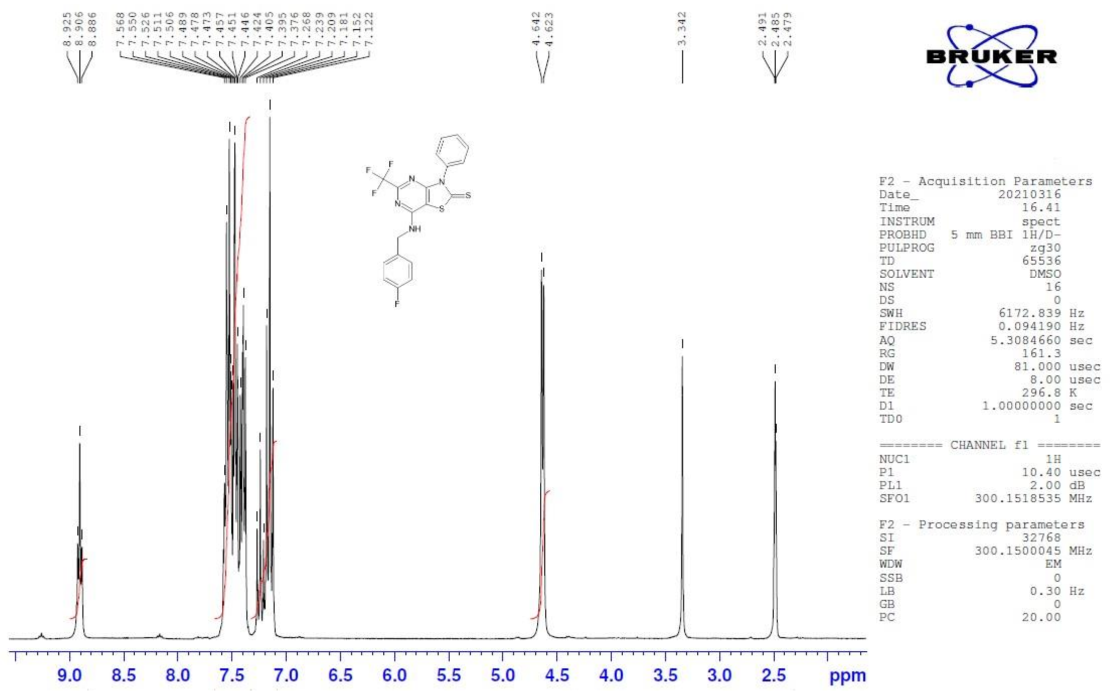

Figure S15. ^{1}HNMR spectrum of compound **4c** (300MHz, DMSO).

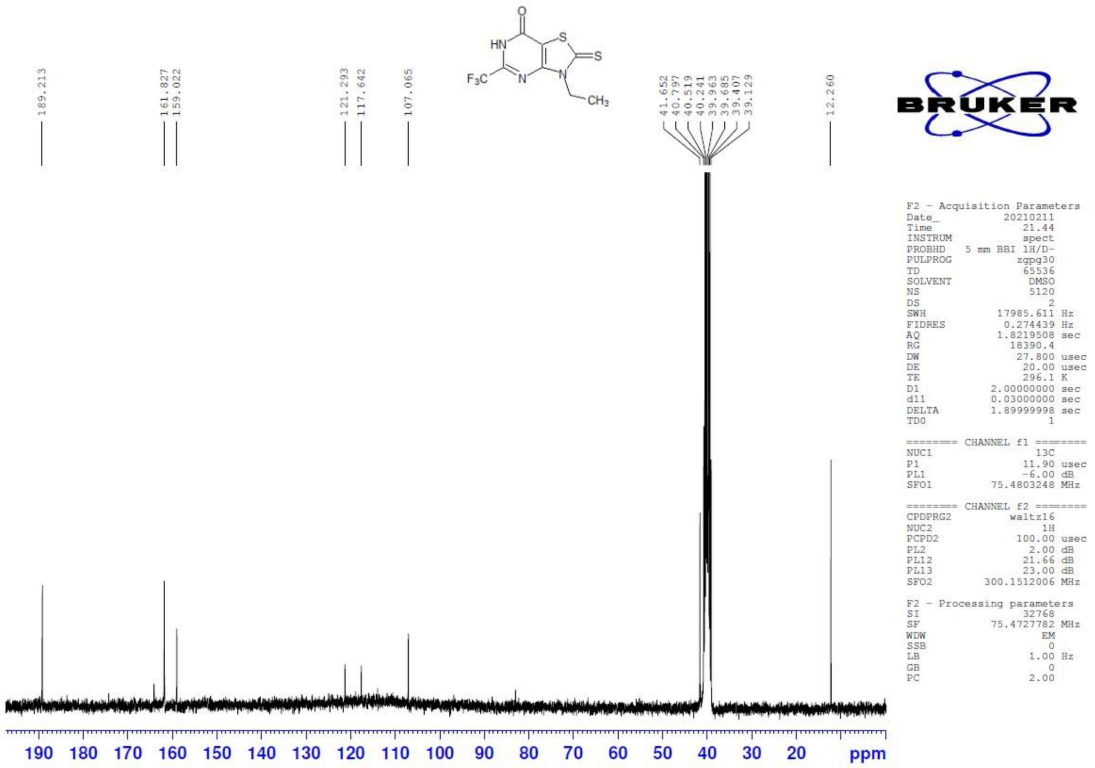

Figure S16. ^{13}CNMR spectrum of compound **2a** (75MHz, DMSO).

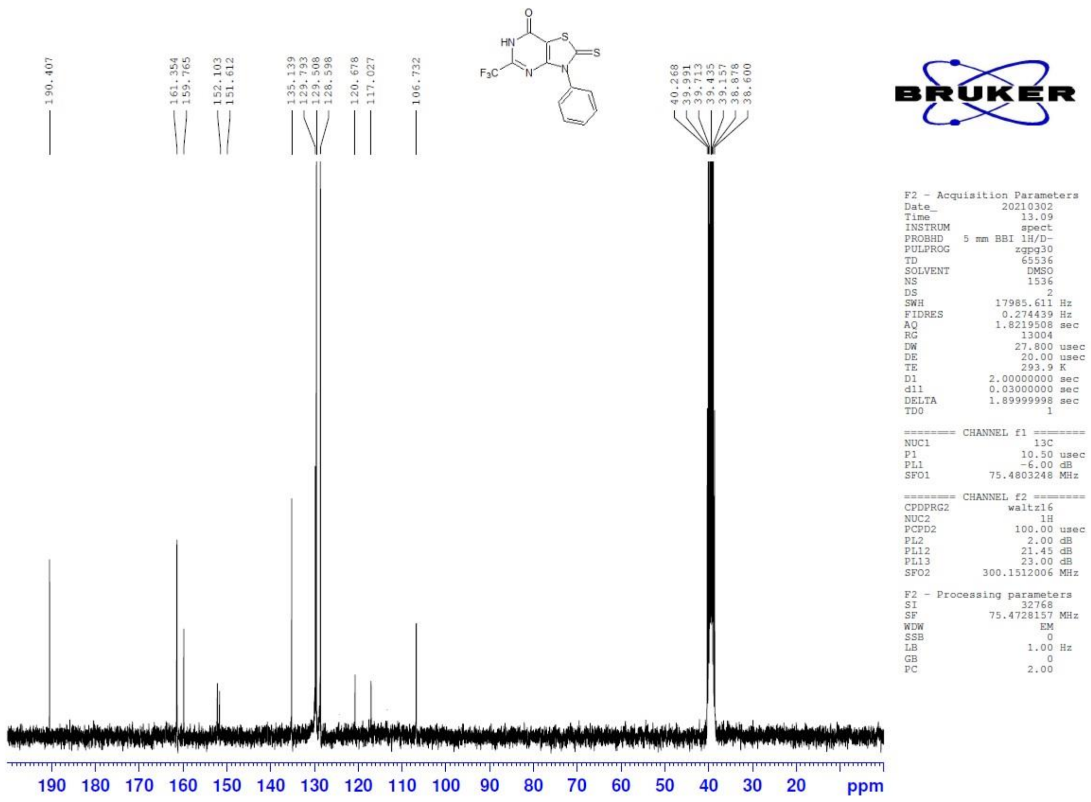

Figure S17. ^{13}CNMR spectrum of compound **2b** (75MHz, DMSO).

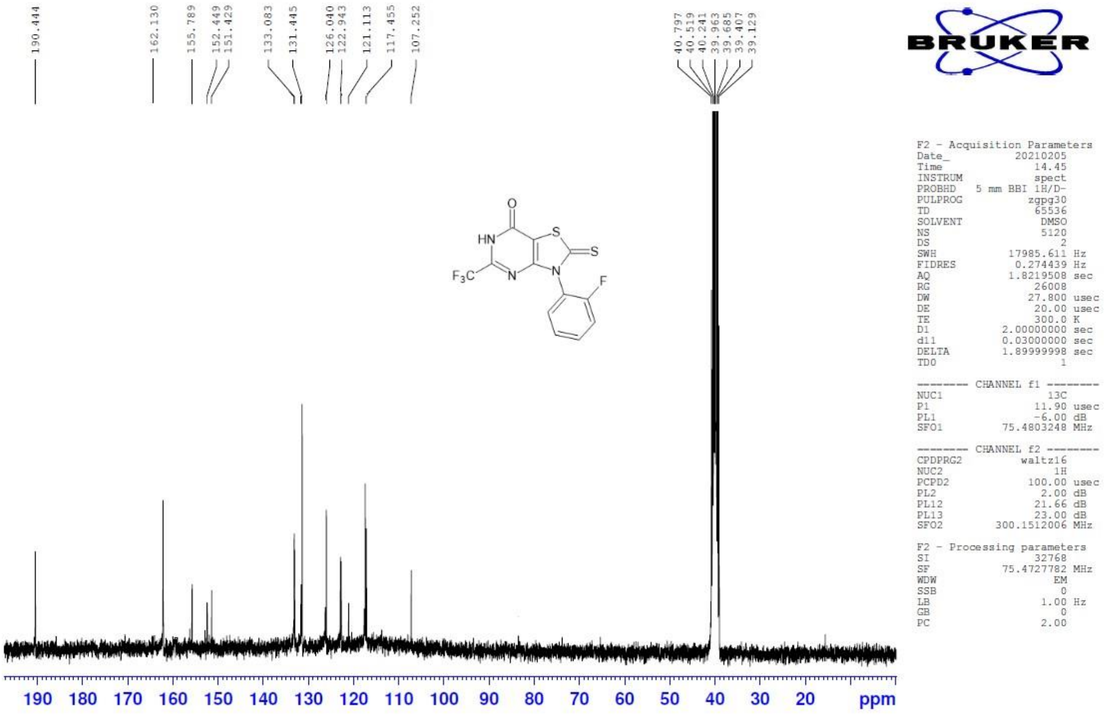

Figure S18. ^{13}CNMR spectrum of compound **2c** (75MHz, DMSO).

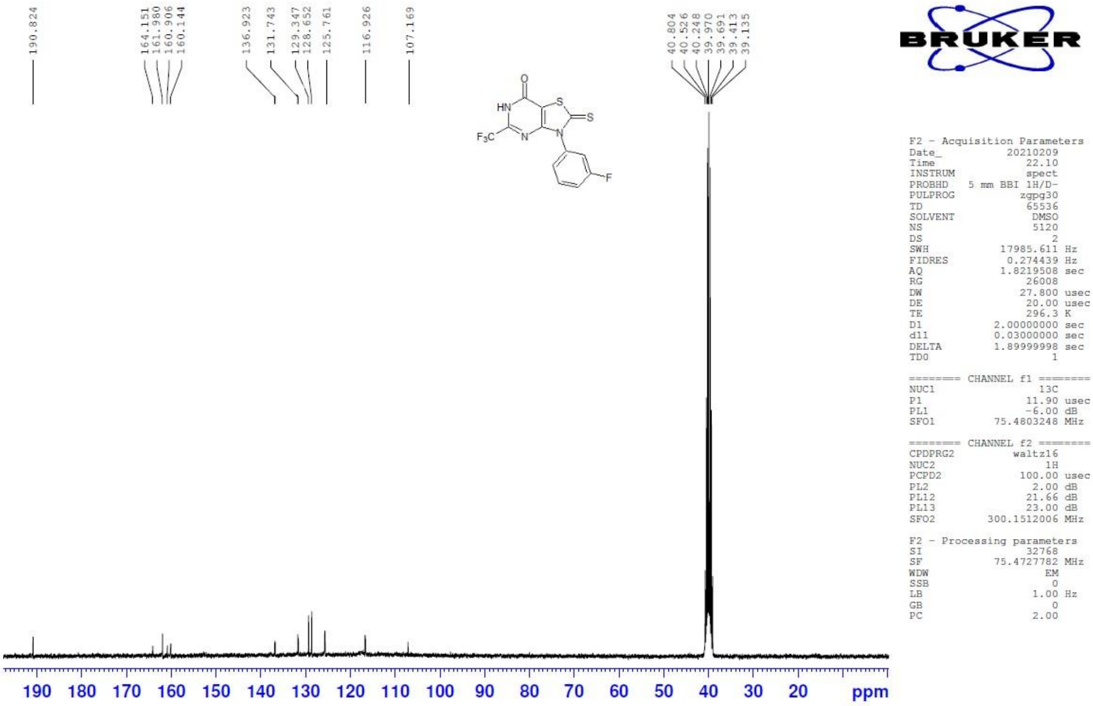

Figure S19. ^{13}CNMR spectrum of compound **2d** (75MHz, DMSO).

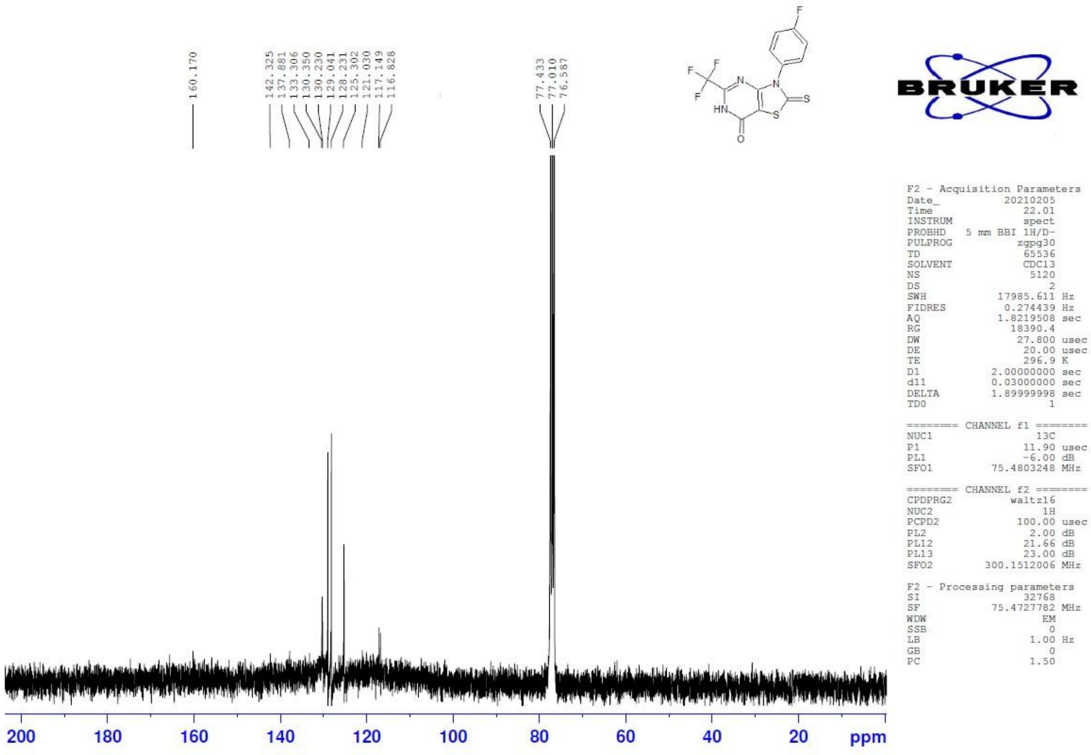

Figure S20. ^{13}CNMR spectrum of compound **2e** (75MHz, $CDCl_3$).

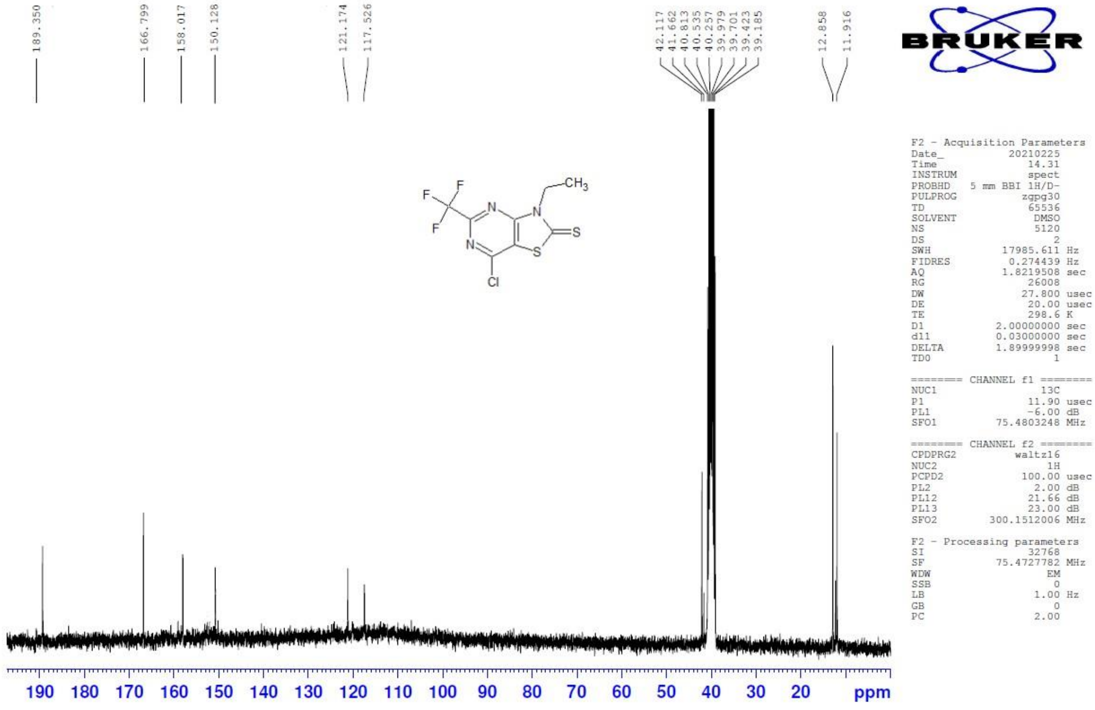

Figure S21. ^{13}CNMR spectrum of compound **3a** (75MHz, DMSO).

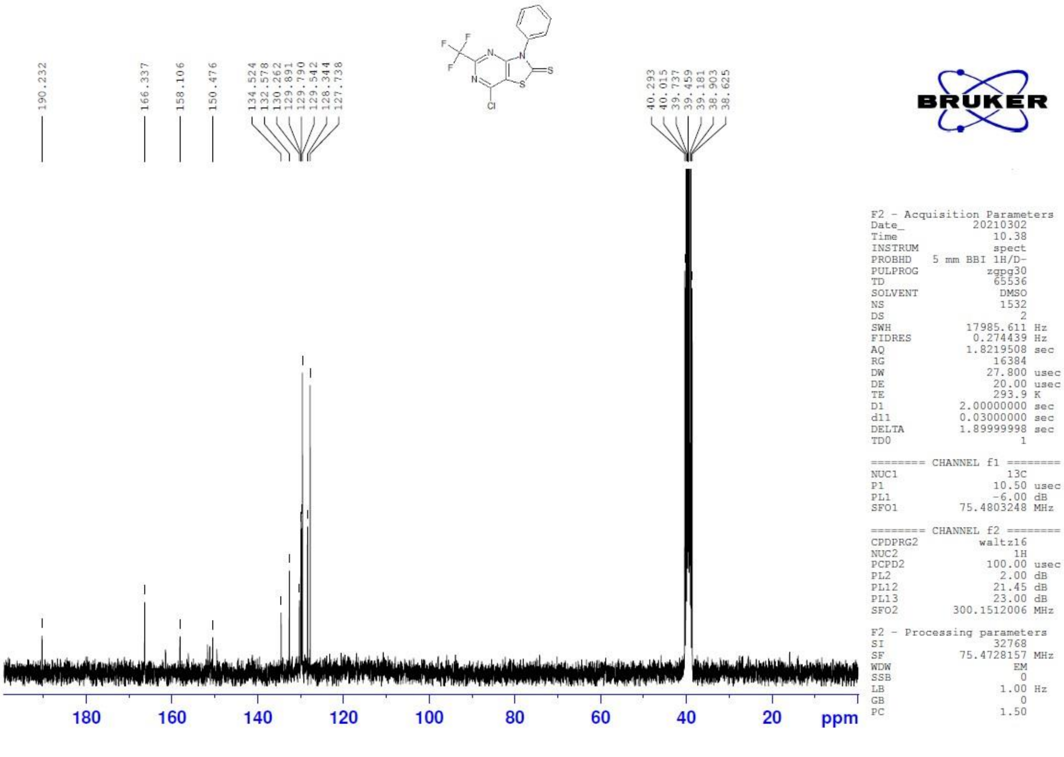

Figure S22. ^{13}CNMR spectrum of compound **3b** (75MHz, DMSO).

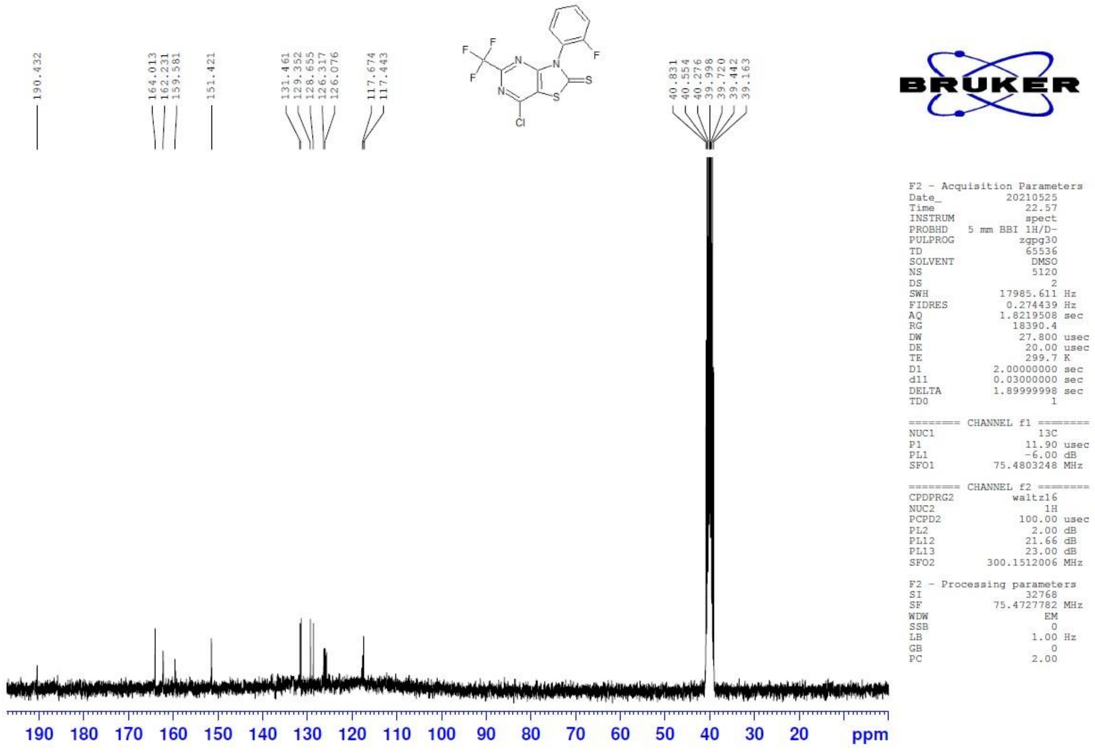

Figure S23. ^{13}CNMR spectrum of compound **3c** (75MHz, DMSO).

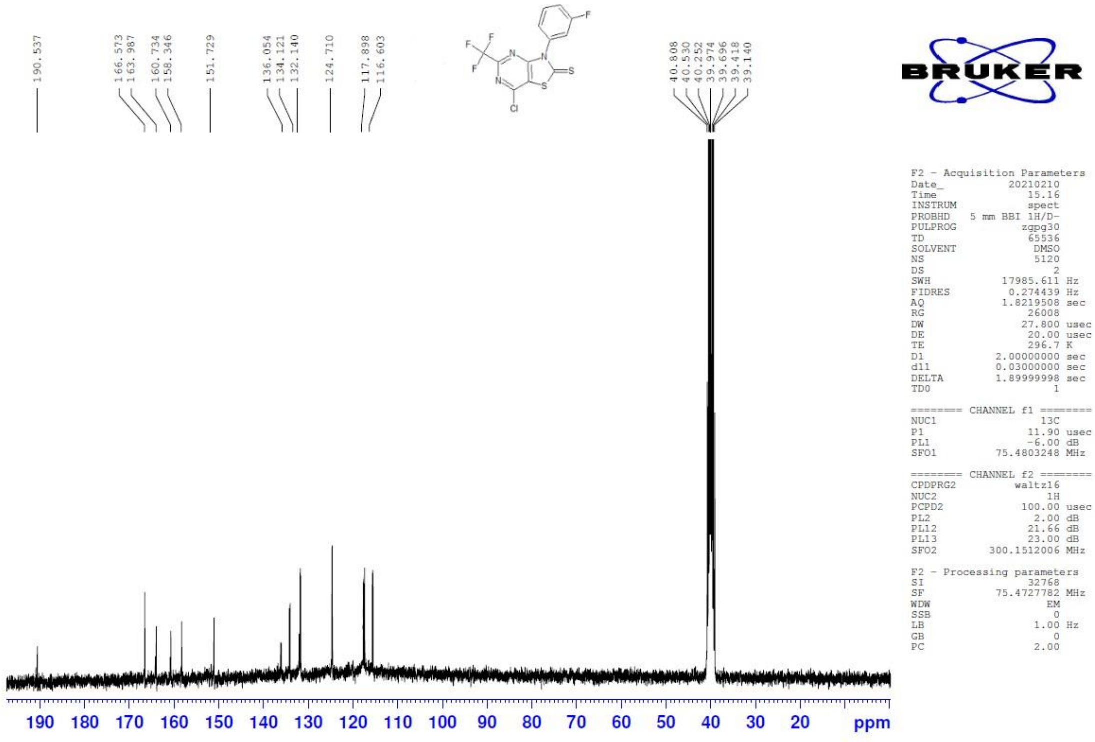

Figure S24. ^{13}CNMR spectrum of compound **3d** (75MHz, DMSO).

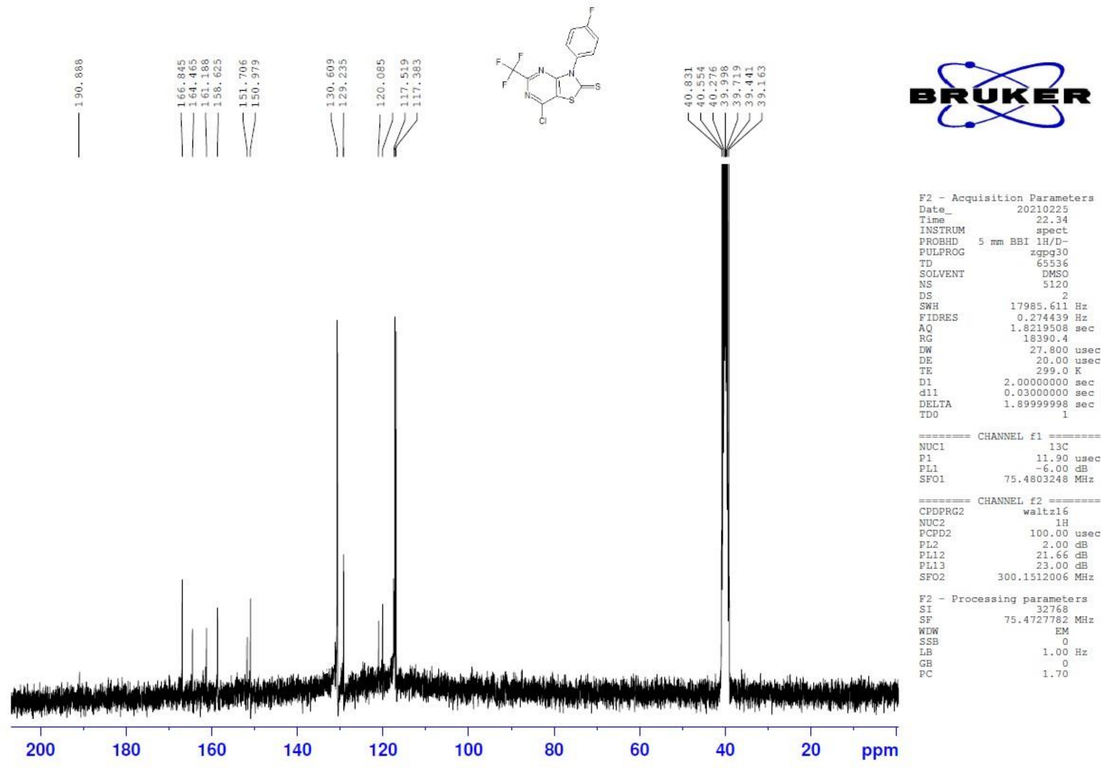

Figure S25. ^{13}CNMR spectrum of compound **3e** (75MHz, DMSO).

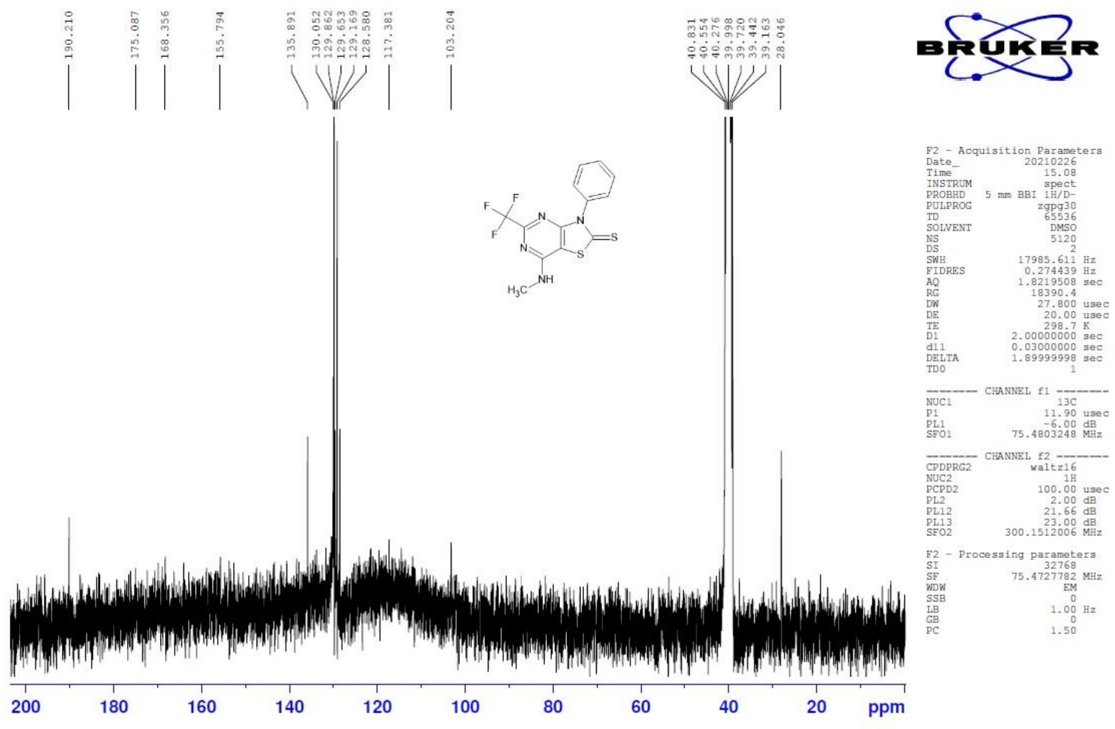

Figure S26. ^{13}CNMR spectrum of compound **4a** (75MHz, DMSO).

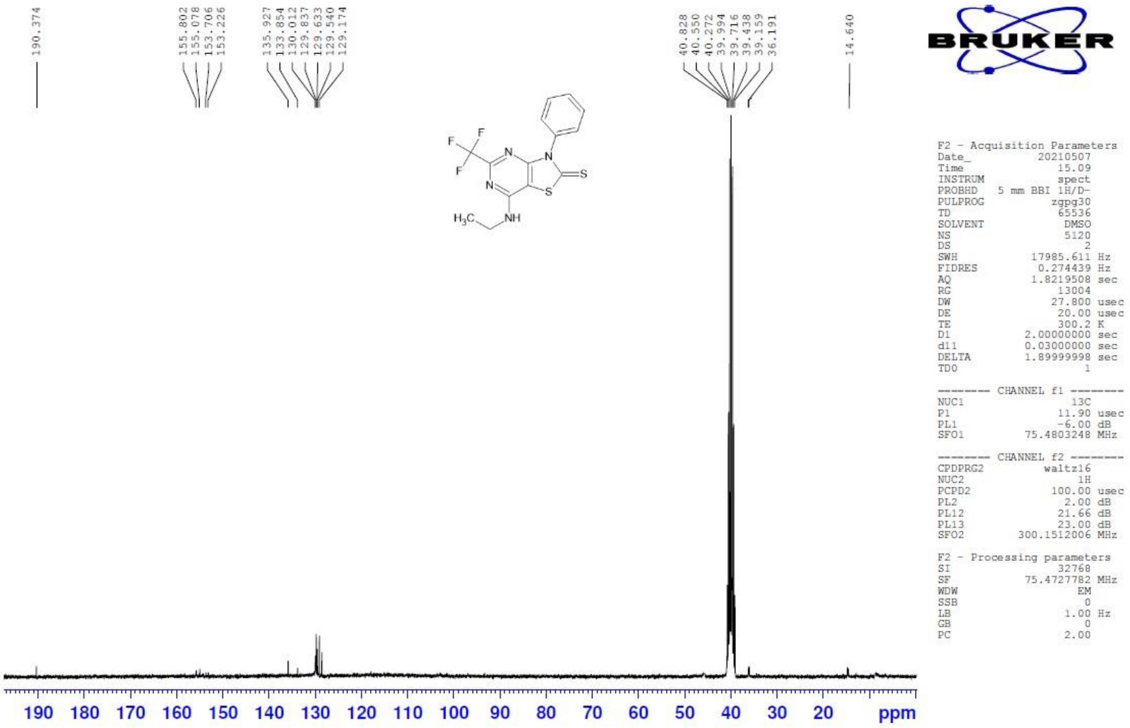

Figure S27. ^{13}CNMR spectrum of compound **4b** (75MHz, DMSO).

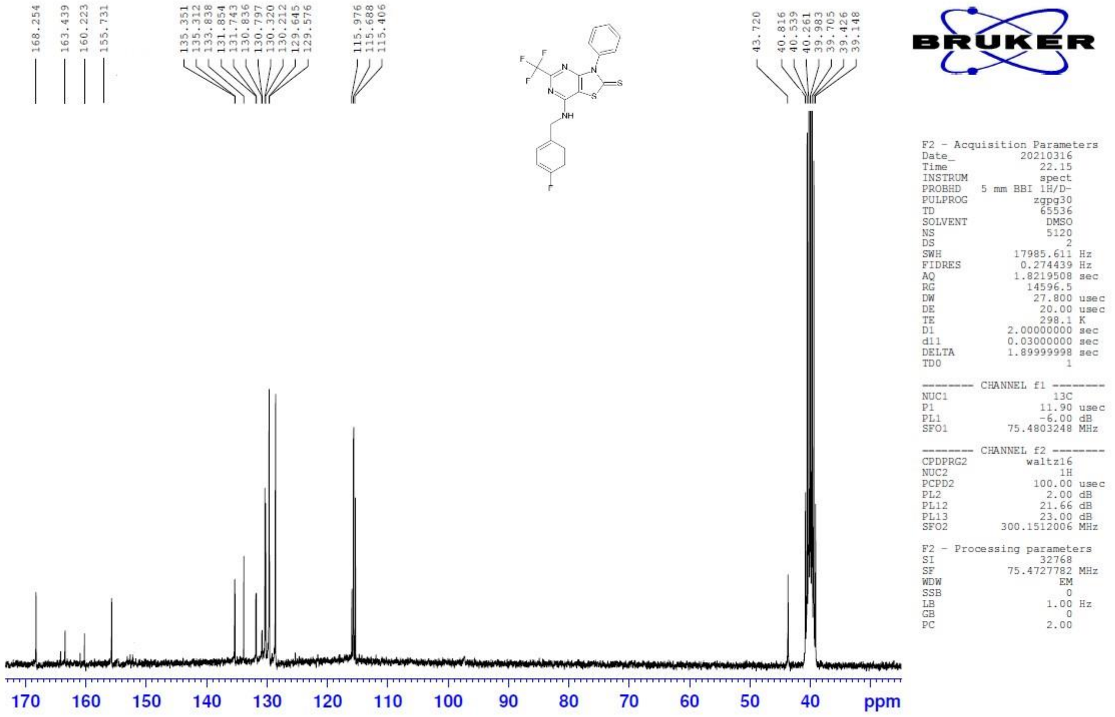

Figure S28. ^{13}CNMR spectrum of compound **4c** (75MHz, DMSO).

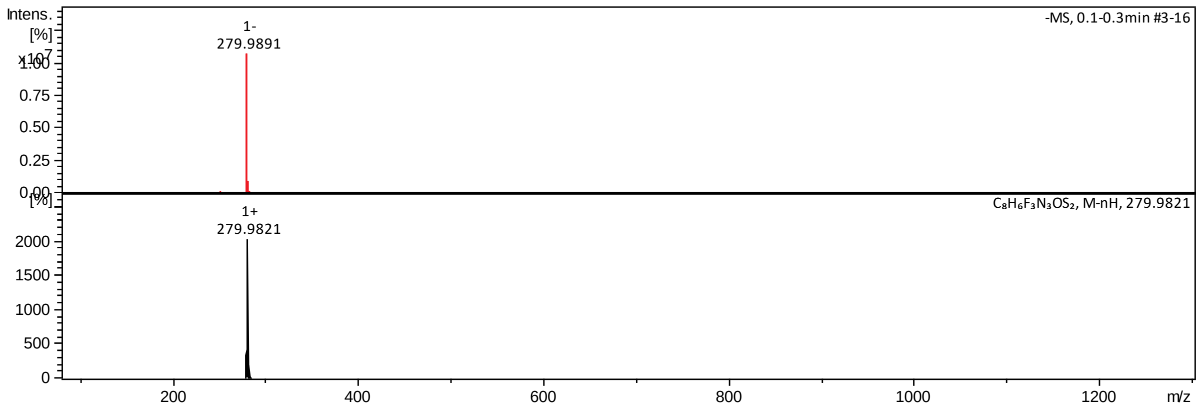

Figure S29. MS spectrum of compound **2a**.

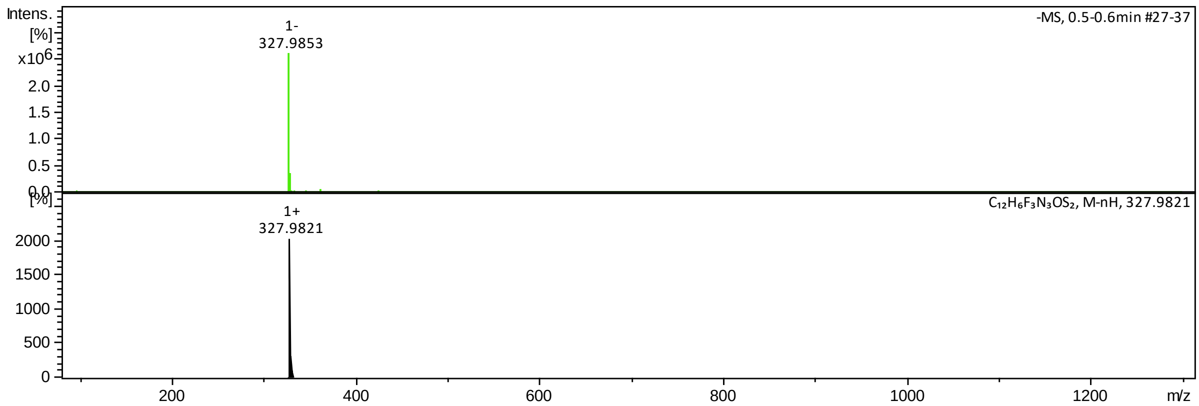

Figure S30. MS spectrum of compound **2b**.

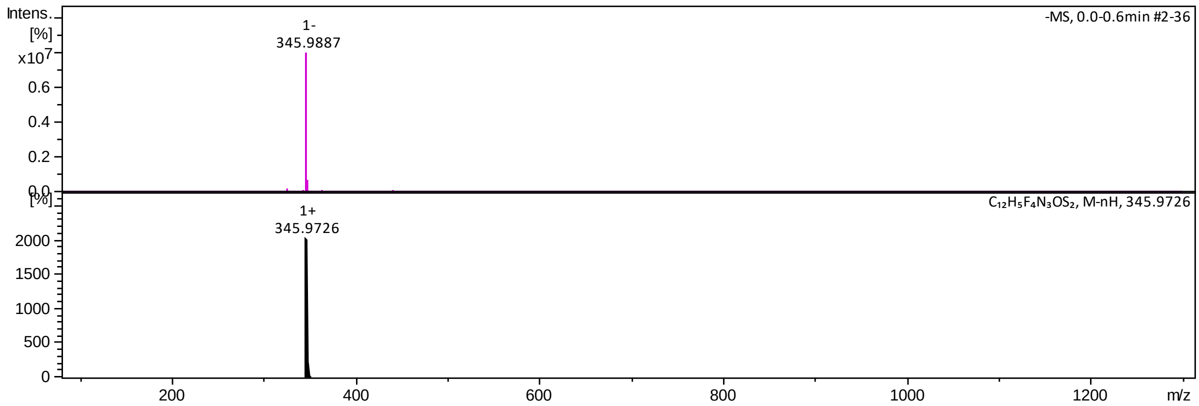

Figure S31. MS spectrum of compound **2c**.

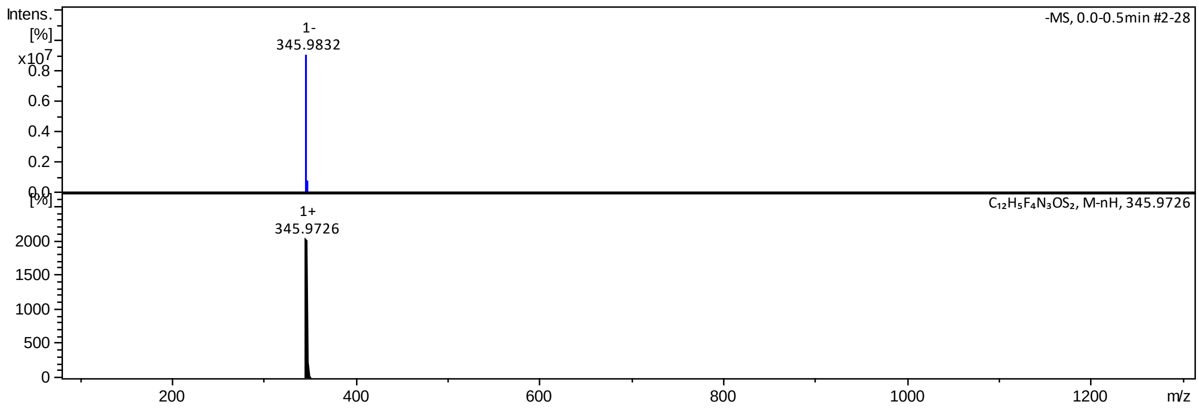

Figure S32. MS spectrum of compound **2d**.

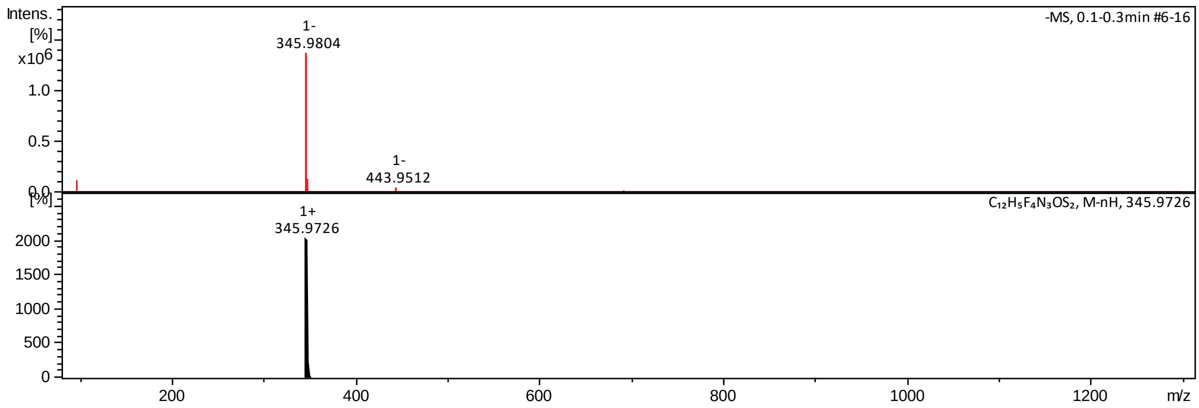

Figure S33. MS spectrum of compound **2e**.

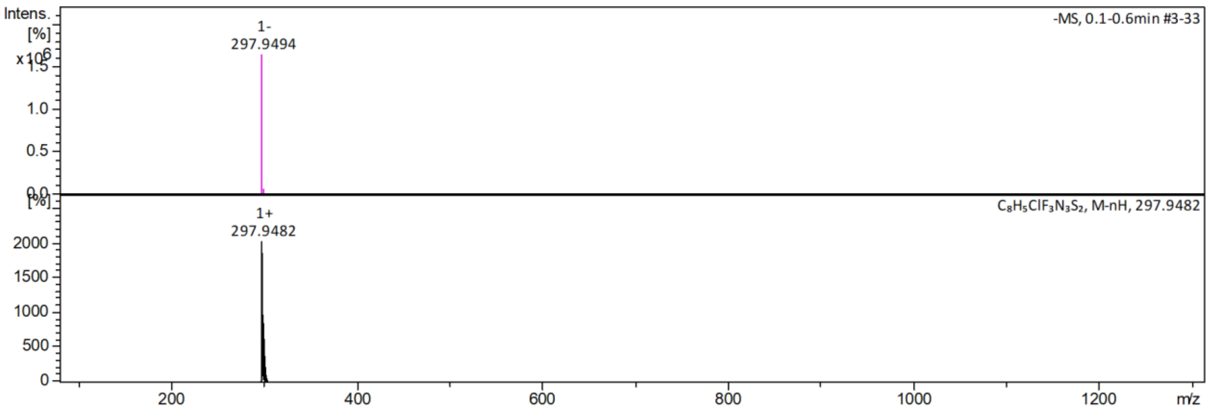

Figure S34. MS spectrum of compound **3a**.

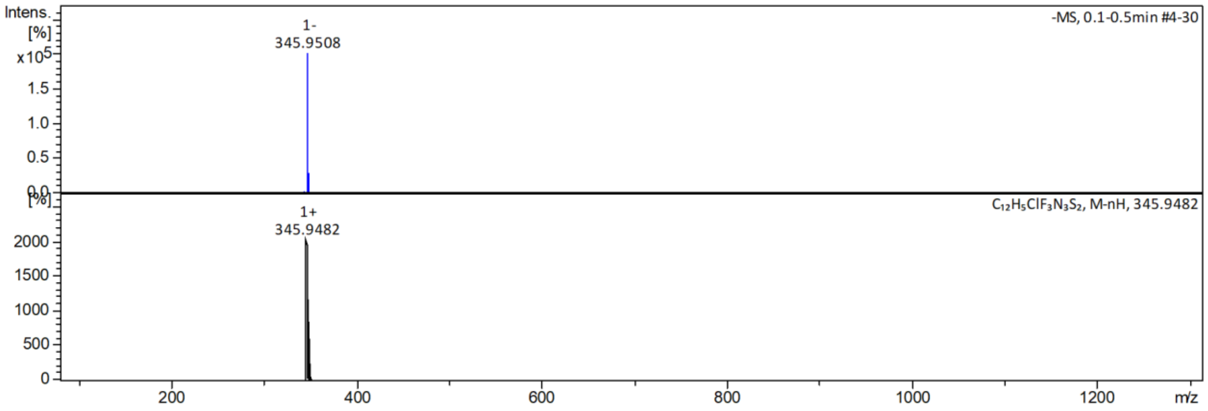

Figure S35. MS spectrum of compound **3b**.

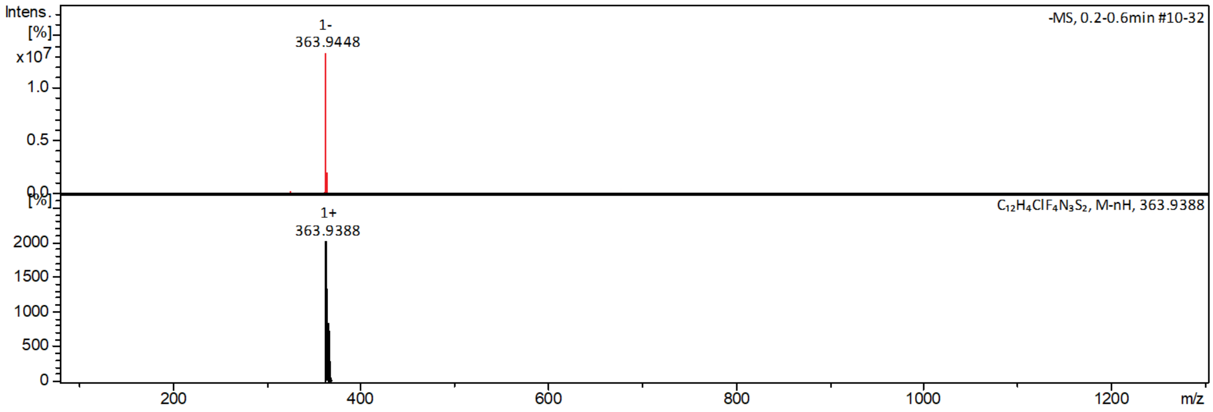

Figure S36. MS spectrum of compound **3c**.

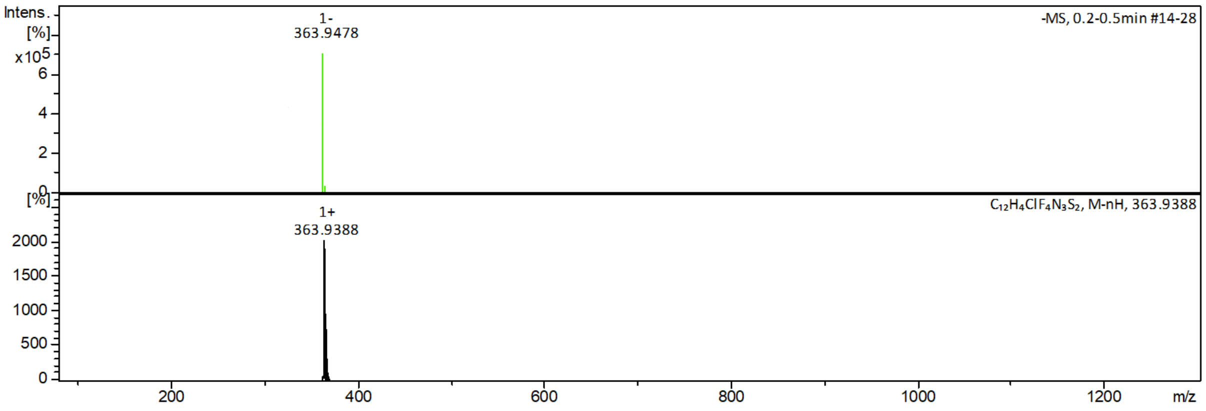

Figure S37. MS spectrum of compound **3d**.

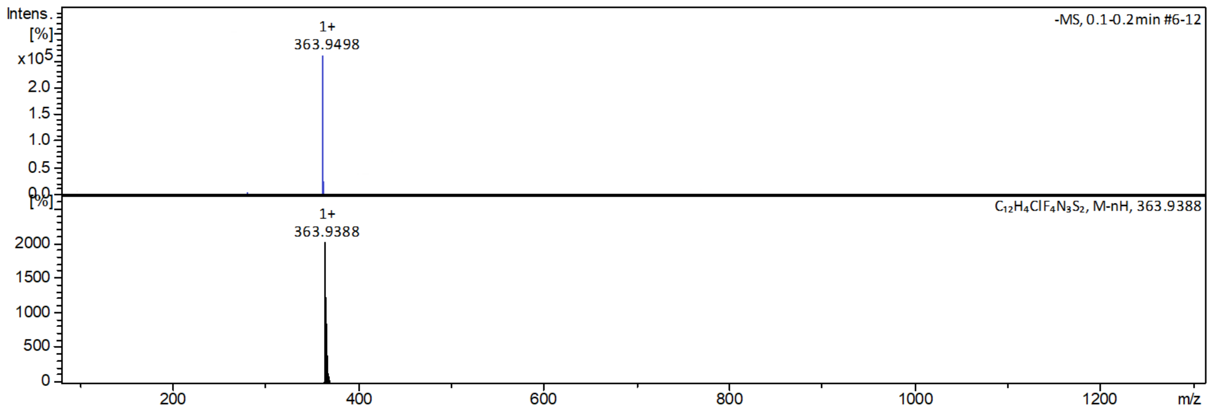

Figure S38. MS spectrum of compound **3e**.

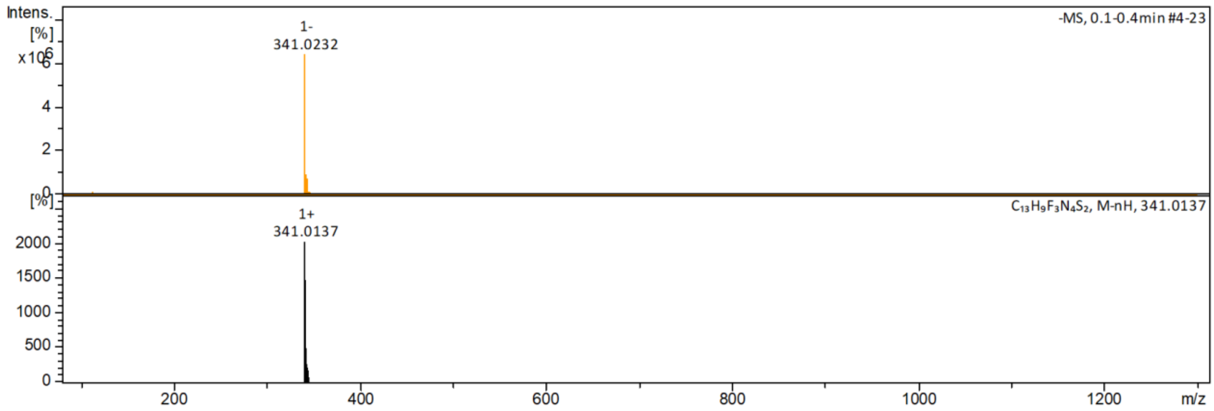

Figure S39. MS spectrum of compound **4a**.

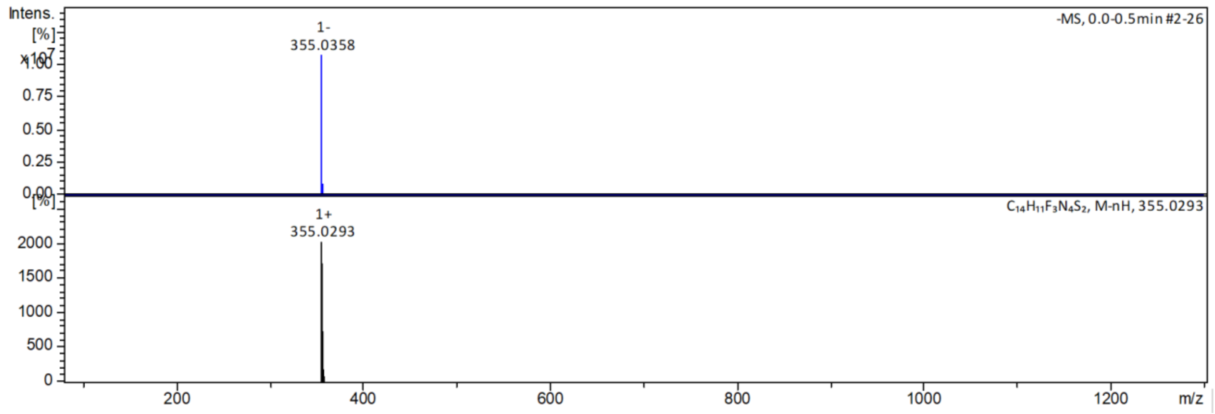

Figure S40. MS spectrum of compound **4b**.

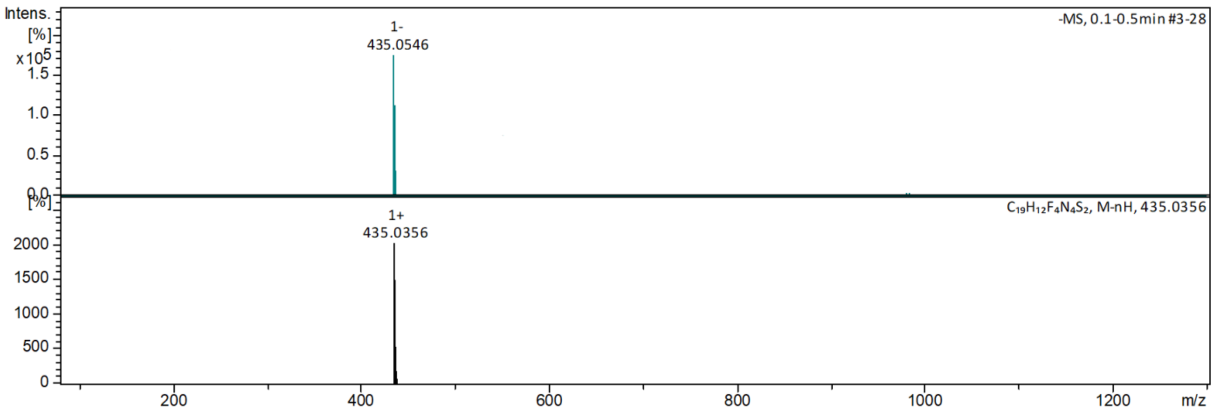

Figure S41. MS spectrum of compound **4c**.

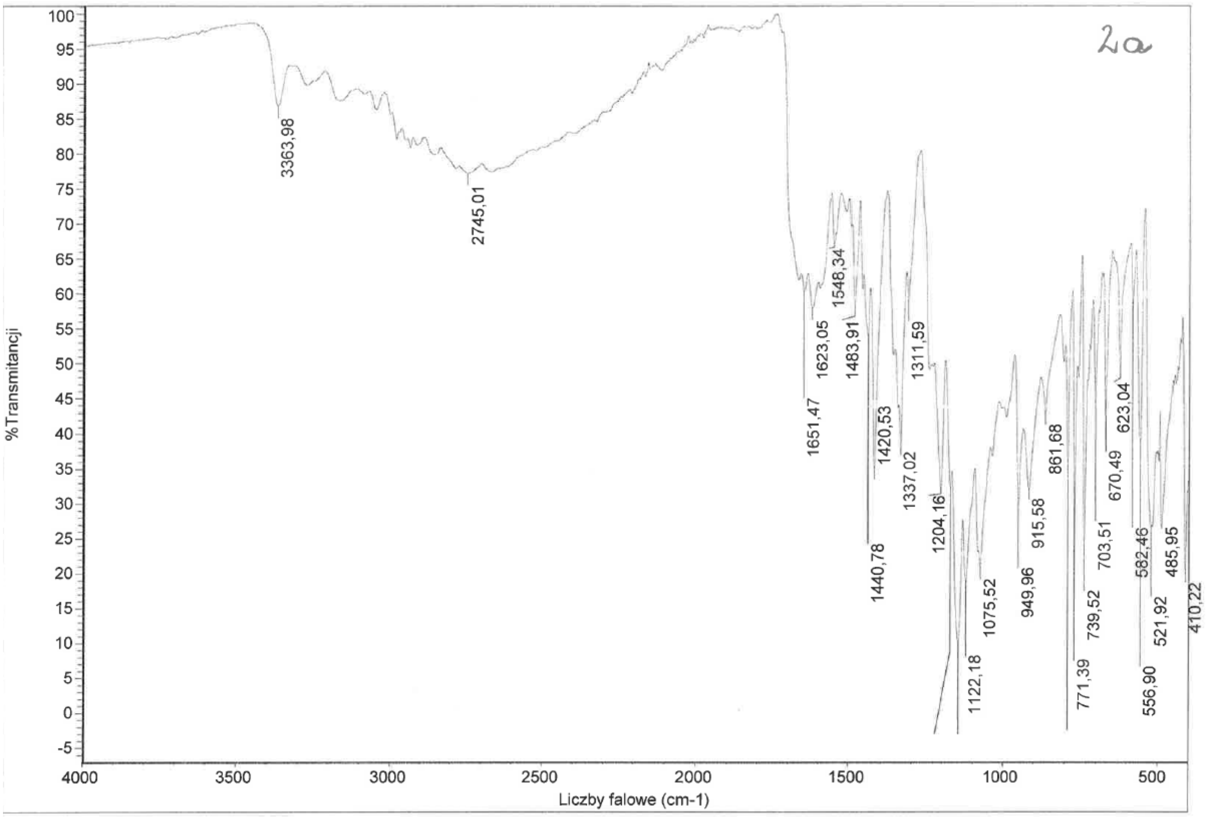

Figure S42. IR spectrum of compound **2a**.

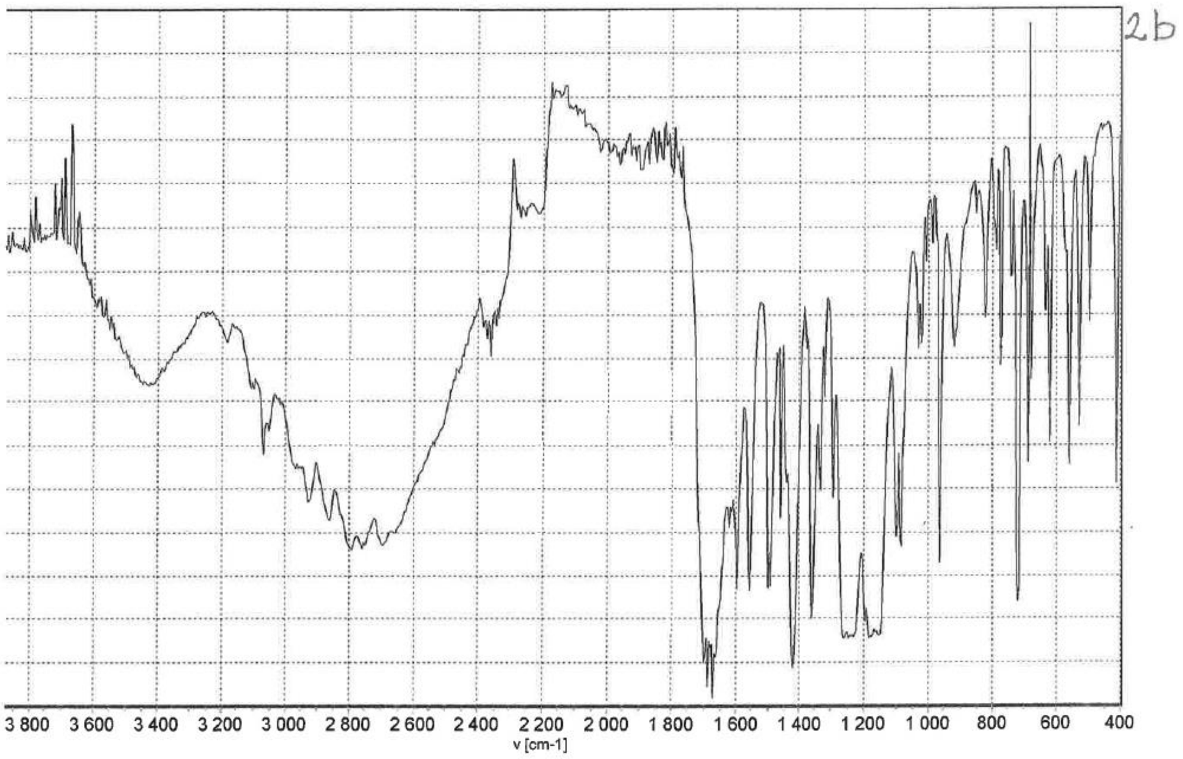

Figure S43. IR spectrum of compound **2b**.

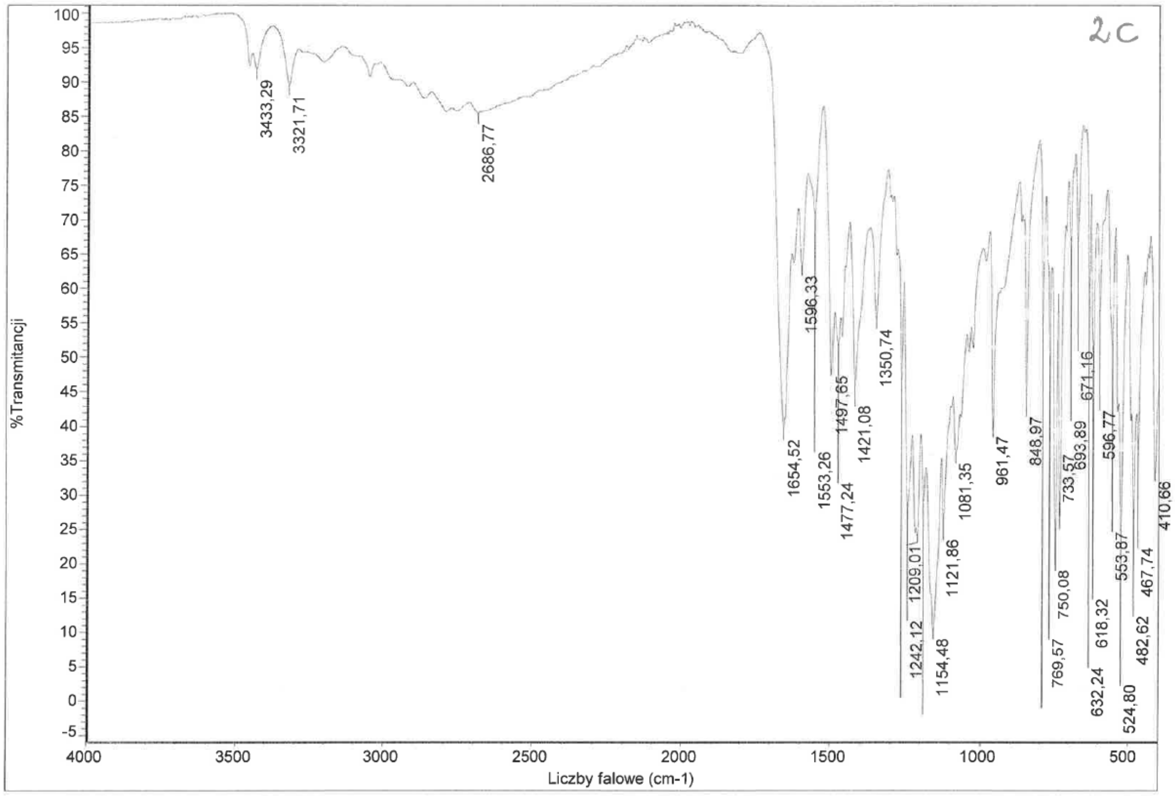

Figure S44. IR spectrum of compound **2c**.

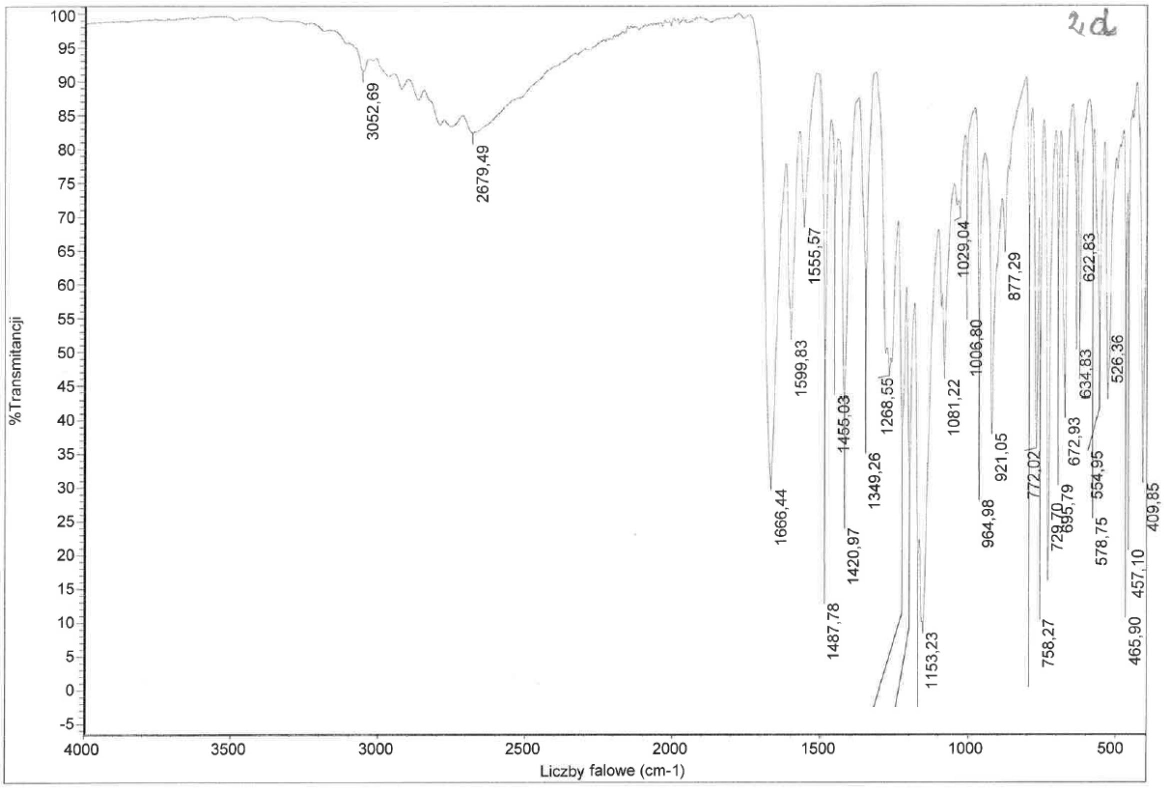

Figure S45. IR spectrum of compound **2d**.

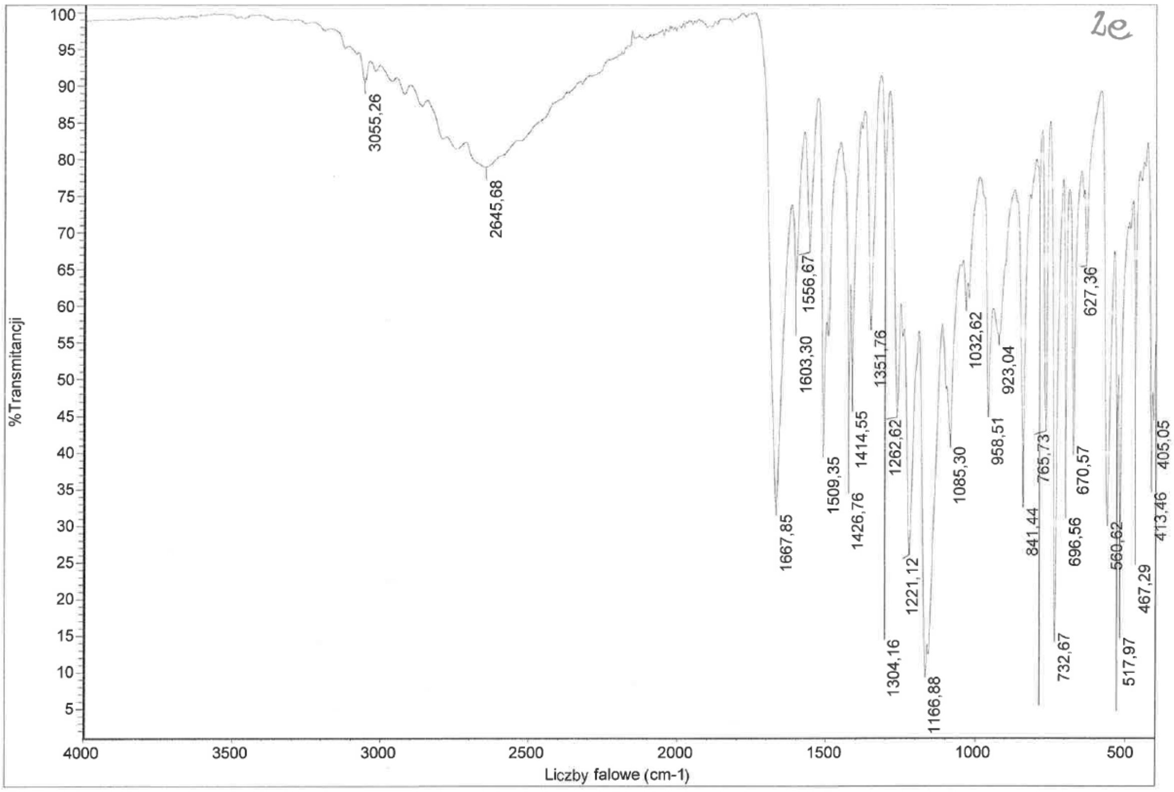

Figure S46. IR spectrum of compound **2e**.

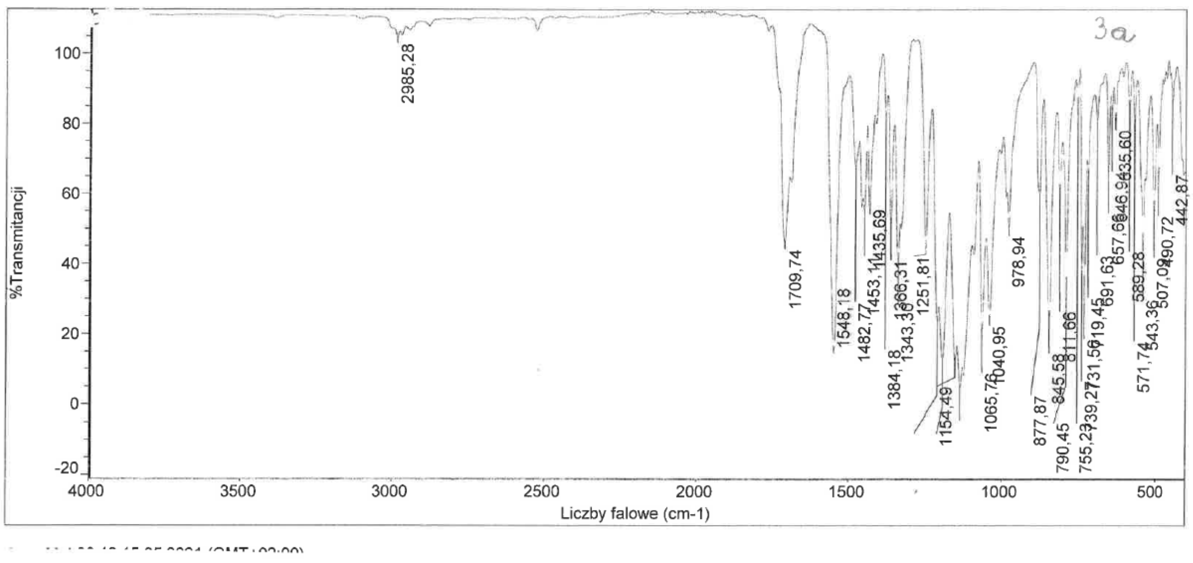

Figure S47. IR spectrum of compound **3a**.

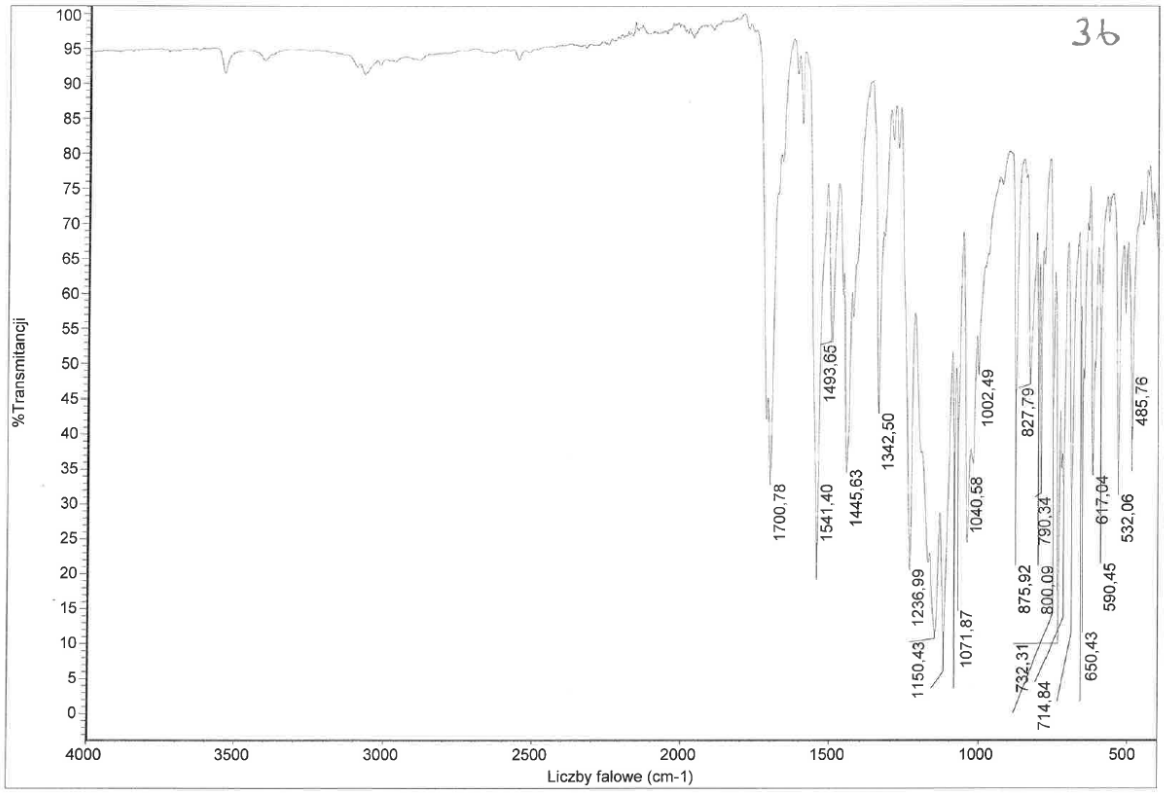

Figure S48. IR spectrum of compound **3b**.

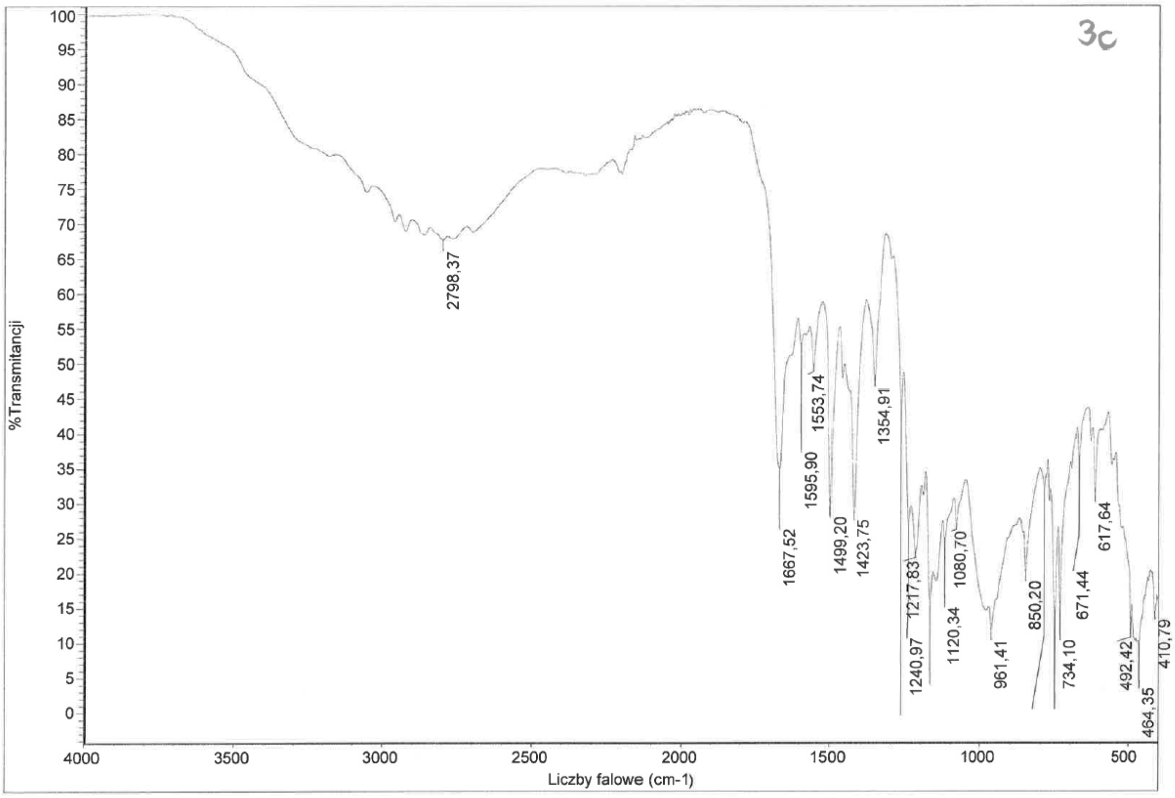

Figure S49. IR spectrum of compound **3c**.

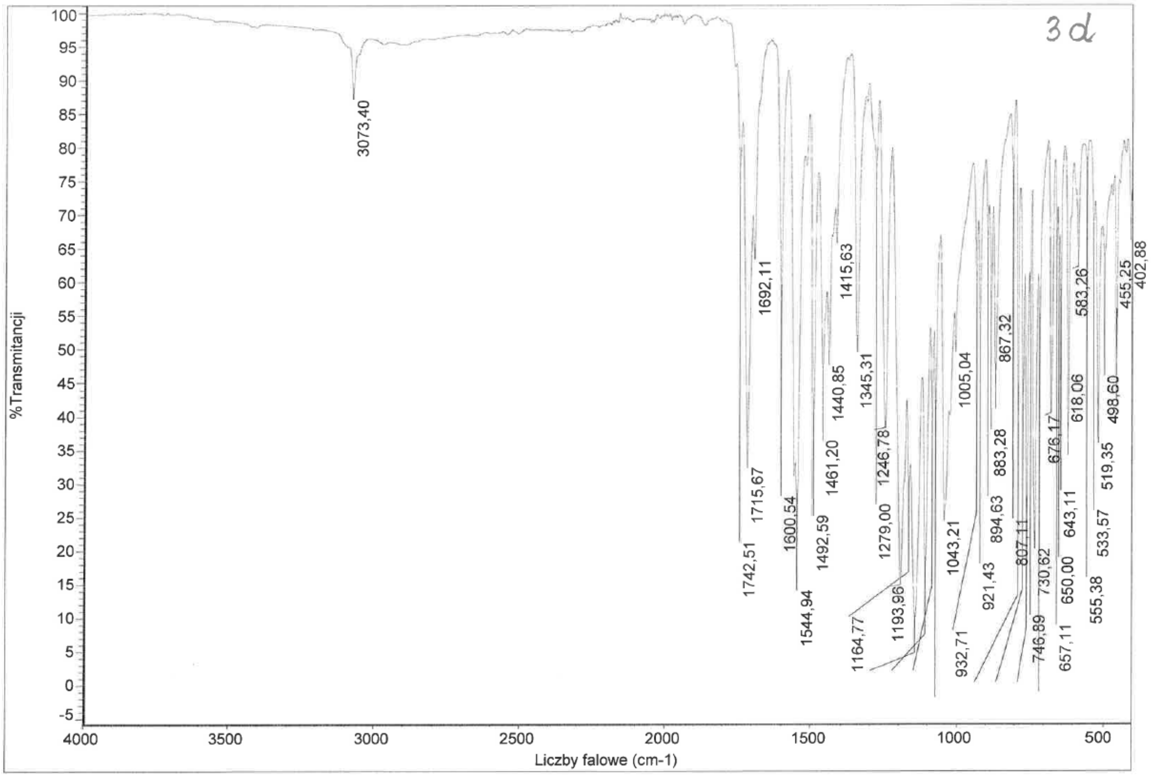

Figure S50. IR spectrum of compound **3d**.

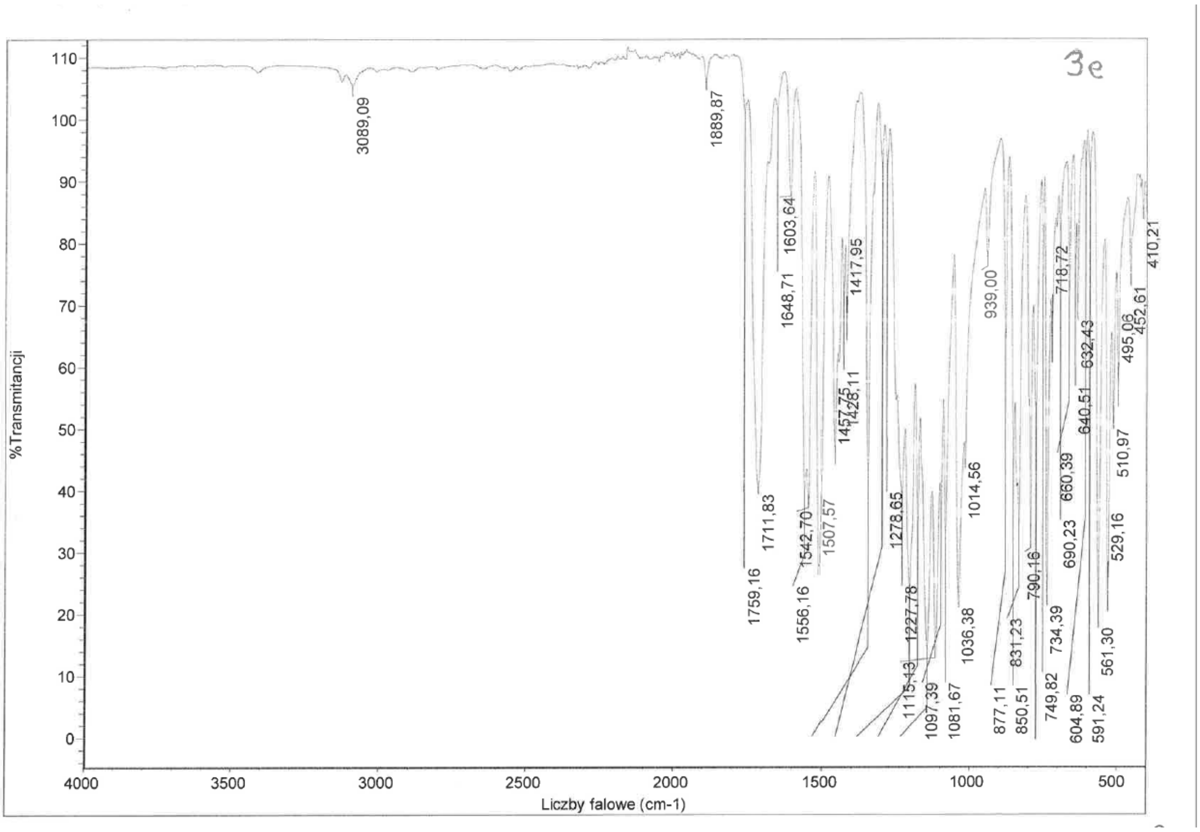

Figure S51. IR spectrum of compound **3e**.

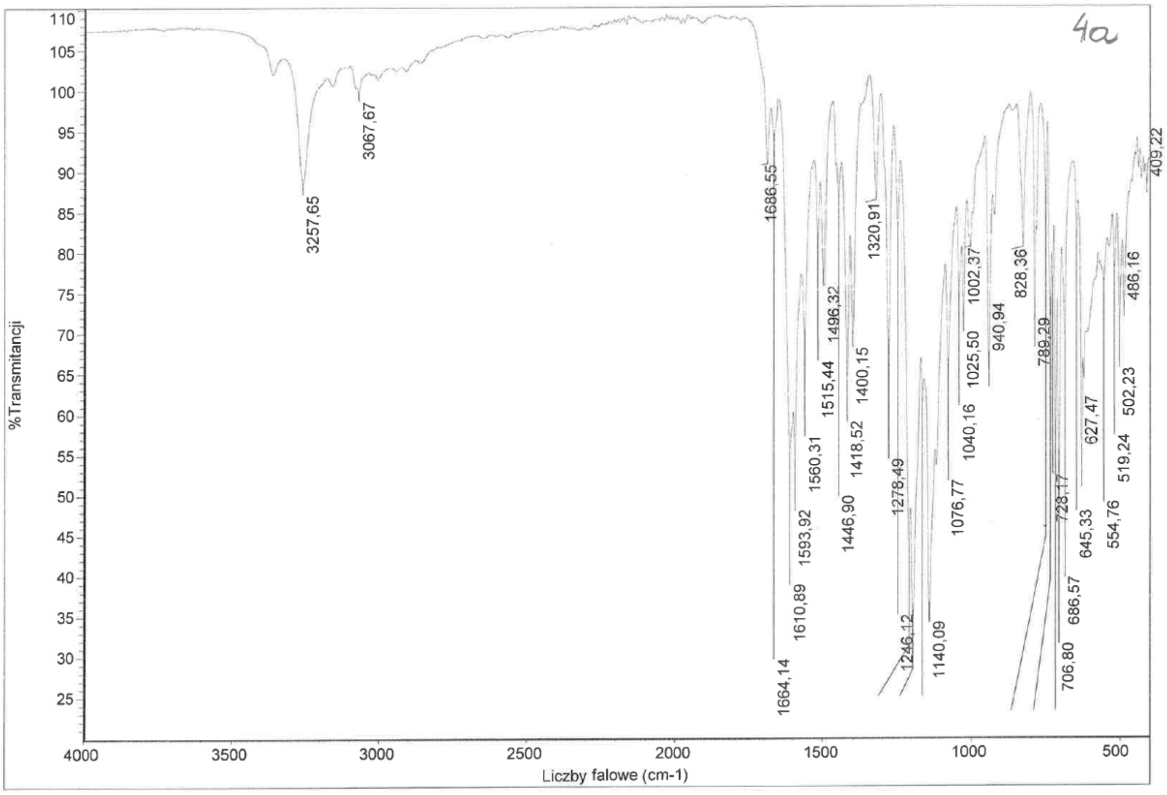

Figure S52. IR spectrum of compound **4a**.

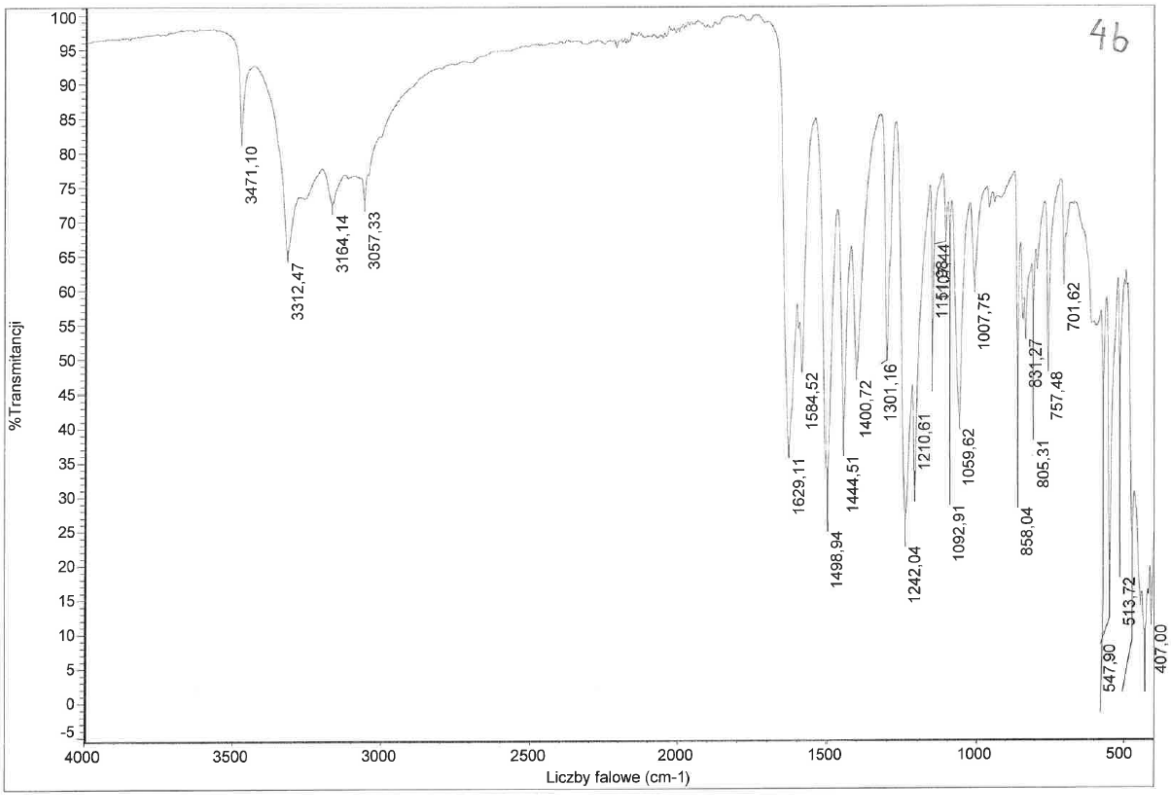

Figure S53. IR spectrum of compound **4b**.

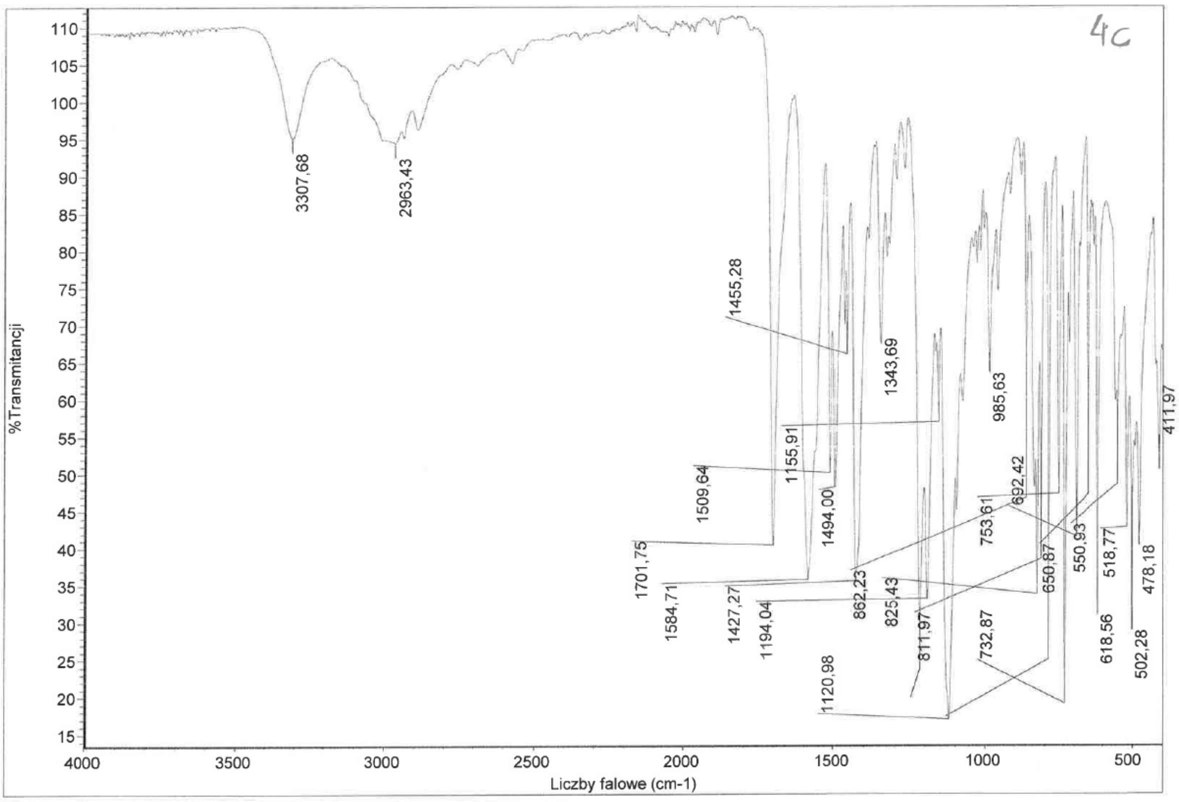

Figure S54. IR spectrum of compound **4c**.

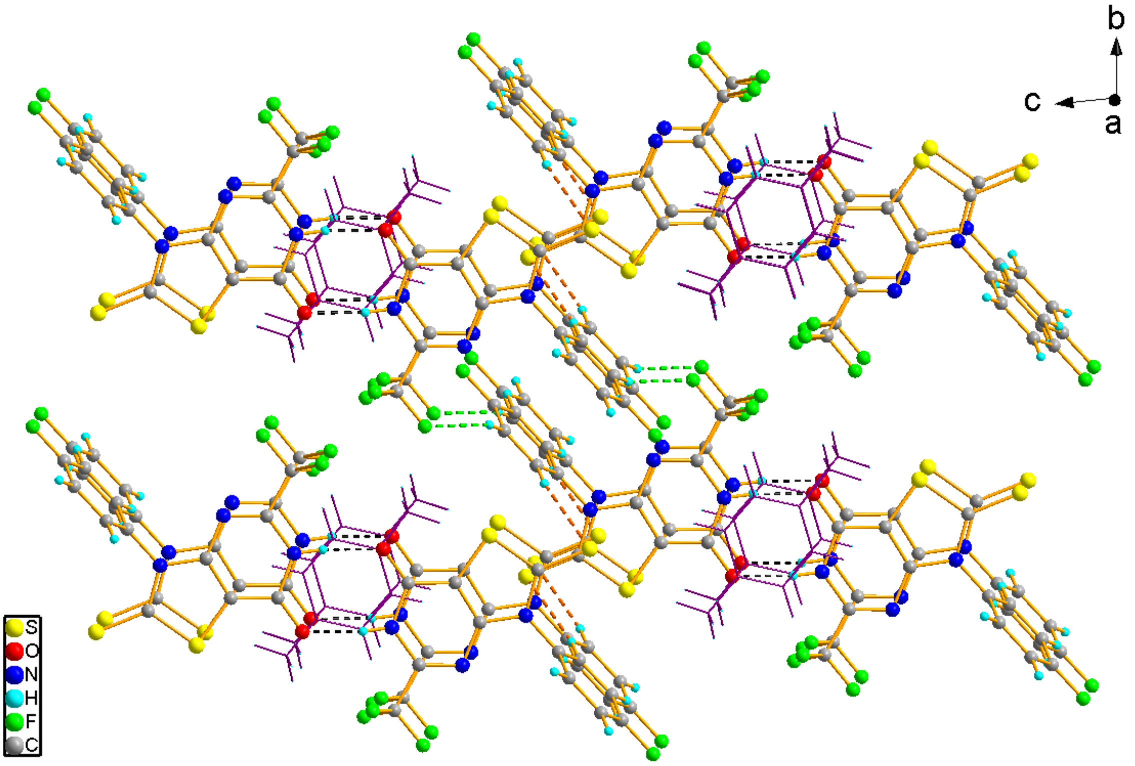

Figure S55. The crystal packing of the **2e** viewed along a axis. Disordered toluene molecules are marked with a purple wire/stick model. Dashed lines (in black) indicate intermolecular interactions, with N—H···O in black, C–H···S in orange and C–H···F in light green.

Table S1. Selected X-ray single-crystal data and structure refinement details of compounds **2e** and **4b**.

	**2e**	**4b**
Chemical formula	2($C_{12}H_5F_4N_3OS_2$), C_7H_8	$C_{14}H_{11}F_3N_4S_2$
M_r (g/mol)	786.75	356.39
Crystal system, space group	triclinic, *P*-1	monoclinic, $P2_1/n$
Temperature (K)	100	100
a, *b*, *c* (Å)	7.217 (2), 9.362 (3), 12.381 (3)	16.228(4), 5.599(2), 17.631(4)
α, β, γ (°)	95.81 (2), 90.55 (2), 99.02 (2)	90, 111.050(5), 90
V (Å^3)	347.46 (17)	1495.1(7)
Z	1	4
λ (Å)	1.54184	1.54184
Crystal size (mm)	0.190 x 0.125 x 0.041	0.207 × 0.034 × 0.021
Crystal colour, shape	yellow, plate	light yellow, needle
D_{calc} (g/cm^3)	1.590	1.583
μ (mm^{-1})	3.45	3.58
F (000)	398	728
2θ range (°)	3.6 - 75.7	3.2-75.5
No of measured, independent and observed [I > 2σ(I)] reflections	16332, 3355, 3066	10153, 3044, 2463
R_{int}	0.030	0.085
Parameters, restraints	336, 0	209, 0
Goof on F^2	1.06	1.04
R_1 [I > 2σ(I)]	0.039	0.075
wR_2 (all data)	0.111	0.215
T_{min}, T_{max}	0.69, 1.00	0.51, 1.00
$\Delta\rho_{max}$, $\Delta\rho_{min}$(e Å^{-3})	0.52, -0.40	1.01, -0.66
CCDC No	2118584	2118585
